# The Centipede Genus *Scolopendra* in Mainland Southeast Asia: Molecular Phylogenetics, Geometric Morphometrics and External Morphology as Tools for Species Delimitation

**DOI:** 10.1371/journal.pone.0135355

**Published:** 2015-08-13

**Authors:** Warut Siriwut, Gregory D. Edgecombe, Chirasak Sutcharit, Somsak Panha

**Affiliations:** 1 Biological Sciences Program, Faculty of Science, Chulalongkorn University, Bangkok, Thailand; 2 Animal Systematics Research Unit, Department of Biology, Chulalongkorn University, Bangkok, Thailand; 3 Department of Earth Sciences, The Natural History Museum, London, United Kingdom; National Cheng-Kung University, TAIWAN

## Abstract

Seven *Scolopendra* species from the Southeast Asian mainland delimited based on standard external morphological characters represent monophyletic groups in phylogenetic trees inferred from concatenated sequences of three gene fragments (cytochrome *c* oxidase subunit 1, 16S rRNA and 28S rRNA) using Maximum likelihood and Bayesian inference. Geometric morphometric description of shape variation in the cephalic plate, forcipular coxosternite, and tergite of the ultimate leg-bearing segment provides additional criteria for distinguishing species. Colouration patterns in some *Scolopendra* species show a high degree of fit to phylogenetic trees at the population level. The most densely sampled species, *Scolopendra dehaani* Brandt, 1840, has three subclades with allopatric distributions in mainland SE Asia. The molecular phylogeny of *S*. *pinguis* Pocock, 1891, indicated ontogenetic colour variation among its populations. The taxonomic validation of *S*. *dawydoffi* Kronmüller, 2012, *S*. *japonica* Koch, 1878, and *S*. *dehaani* Brandt, 1840, each a former subspecies of *S*. *subspinipes* Leach, 1814 sensu Lewis, 2010, as full species was supported by molecular information and additional morphological data. Species delimitation in these taxonomically challenging animals is facilitated by an integrative approach that draws on both morphology and molecular phylogeny.

## Introduction

Several lines of evidence suggest that Southeast Asia, one of world’s biodiversity hotspots, might be a migration corridor for animals [1]. Currently, Southeast Asian biodiversity is classified as two zoogeographical sub-regions, Indochina and Malesia [2]. Associated with the geographical richness of this area, cryptic speciation has been reported in several endemic groups, including molluscs [3], decapods [4], insects [5], fish [6] and amphibians [7], and has been attributed to allopatric and sympatric speciation, both on land and on islands [8].

Molecular phylogeny has emerged as a powerful tool for evolutionary studies across the Tree of Life [9, 10]. Drawing on molecular techniques, the classification and evolutionary history of organisms has been revised and refined [5, 7, 11–16]. Recent studies on a global scale of phylogenetic biogeography of evolutionarily ancient animal taxa such as onychophorans [17, 18] and centipedes in the order Scutigeromorpha [19, 20] revealed insights on genetic affinity and evolutionary history through past geological events. Moreover, smaller scale phylogeographic studies have proven valuable for exploring spatial and phylogenetic patterns in the diversification in some groups of centipedes, such as the geographically restricted Australasian order Craterostigmomorpha [13]. However, this approach to historical biogeography has not yet been applied to any centipedes in Southeast Asia.

The genus *Scolopendra* Linnaeus, 1758 comprises common large scolopendromorph centipedes that are distributed worldwide, especially in tropical territories, and nowhere more than in Southeast Asia [21]. In the Old World, *Scolopendra* consists of 42 nominal species [22]. However, because of its scattered distributional records, the fine details of the distributional range of several species are still unknown and need to be revised. Some *Scolopendra* species, such as *S*. *morsitans* Linnaeus, 1758, and *S*. *subspinipes* Leach, 1814 [23], have been interpreted as widespread species and recognized as introduced by human transportation because of their habitat preferences, and in some cases their commercial usage [24]. Recent studies on molecular phylogeny of Mediterranean *S*. *cingulata* (Latreille, 1829) [25] explored the genetic affinity between adjacent populations and also interpreted the evolutionary history of its geographical distribution in the past in relation to geological events [26, 27].

However, there remains considerable morphological ambiguity among members of *Scolopendra*, a problem that has been discussed in relation to a shortage of informative taxonomic characters that can be used for species delimitation [28]. Moreover, the validity of some members needs to be reassessed because their purportedly diagnostic characters show a high degree of geographic variability [22]. The status of various nominal taxa within *Scolopendra* as either full species, as subspecies, or as variants within species that do not warrant taxonomic recognition is especially acute in *S*. *subspinipes*. This nominal species had long been assigned as many as seven subspecies [29], the status of which has been repeatedly debated [30]. Recently, geometric and meristic morphometrics has been proposed as a method for helping to clarify shape problems in some centipede groups such as Scutigeromorpha [31, 32] and Scolopendromorpha, including species of *Scolopendra* [33]. These studies invite the application of such techniques for attempting to resolve taxonomic problems in other groups.

According to many previous taxonomic studies, colouration patterns in the body of *Scolopendra* vary among populations from different geographical regions [34, 35]. However, there are still limited data for evaluating this character for its taxonomic value, and its relationship to phylogenetic and phylogeographic patterns are all but unexplored.

In this study, seven *Scolopendra* species from the Southeast Asian mainland have been collected and examined. The concatenated sequences of three gene fragments, specifically the barcode region of cytochrome *c* oxidase subunit 1, 16S mitochondrial DNA, and partial 28S nuclear ribosomal DNA, have been used to construct phylogenetic trees and perform a biogeographic study. Geometric morphometrics has been implemented to record shape variation in three selected features using landmark methods. These features are the forcipular coxosternite, the cephalic plate, and the tergite of the ultimate leg-bearing segment. Moreover, colouration patterns in some *Scolopendra* species have been recorded at both the species and population levels, allowing this variation to be mapped on phylogenetic trees. We apply these different data sources and analytical approaches together with traditional external morphological characters to the question of species delimitation in *Scolopendra*.

## Materials and Methods

### Specimen collection and identification

Centipede specimens were collected from both natural and urban habitats through the course of biodiversity surveys in Thailand and adjacent countries under grants to SP since 2010. Permission to enter remote and natural reserve areas was granted by The Plant Genetic Conservation Project under the Initiative of Her Royal Highness Princess Maha Chakri Sirindhorn (grant proposals 2010–2015). During field surveys, some individuals were photographed with either a Nikon D700 or D90 camera equipped with a Nikon AF-S DX Micro-Nikkor 40 mm f/2.8G lens and two Nikon SB 600 Speedlight flash sources to record their living colouration pattern. Collected samples were relaxed with 50% ethanol concentration mixture for 5–20 minutes and then transferred to a higher ethanol concentration for setting their posture for photography. Species identification was made based on previous literature on scolopendrid taxonomy, i.e. studies by Attems [29, 36], Chao [37], Lewis [22, 38, 39] Shelley et al. [24, 35] Schileyko [40–42], Koch [34], Kronmüller [30, 43] and Verhoeff [44]. Terminology applied to taxonomic characters used the standardized nomenclature for centipede morphology by Bonato et al. (2010) [45]. All taxonomic characters were observed under an Olympus stereo microscope connected with a montage imaging system operated under the Cell’D program. All examined materials are housed at Chulalongkorn University, Museum of Zoology, Bangkok, Thailand. Molecular samples are kept in absolute ethanol at -40 degrees Celsius to prevent DNA degradation.

### DNA extraction, amplification and sequencing

Sixty *Scolopendra* samples were dissected to separate tissue from locomotory legs for DNA extraction. The commercial Geneaid DNA extraction and Nucleospin purification kits were used for harvesting genomic DNA. Genomic DNA yields were determined using DNA quantification loading dye blue, loaded in 1X TBE-Agarose gel and run under a 135 V electrical gradient impulse for 15 minutes. Standardized conditions of PCR amplification were edited based on previous molecular works on centipede phylogeny as follow; Edgecombe and Giribet, 2008 [13], Joshi and Karanth, 2011; 2012 [46, 47], Murienne et al. 2010; 2011 [48, 49], Vahtera et al. 2012, 2013 [50, 51] and Siriwut et al. 2015 [52]. Three partial gene fragments were used in this study; the barcode region of cytochrome *c* oxidase subunit I (COI), 16S ribosomal DNA (16S rRNA), and 28S nuclear ribosomal DNA (28S rRNA).

The PCR mixture consisted of the following: 0.6–1 μl of DNA template, 2.5 μl of forward and reverse primers, 25 μl of Ultra-Pure *Taq* PCR Master Mix combined with ruby and emerald loading dye and 18 μl of ddH_2_O. All gene fragments were amplified using the selected primers for each region as follow: COI was assembled using forward universal primers for COI for animal phylogeny LCO1490 [53] and the modified reverse primer for centipede phylogeny HCOoutout [49, 54], 16S rRNA used primers 16Sa and 16Sb [51, 55], and 28S rRNA was amplified by 28SF4 and 28SR5 [56].

All PCR mixtures were activated by an Eppendorf Mastercycler Pro S machine with manual and gradient functions. The COI, 16S and 28S gene amplifications were performed under standard conditions of PCR reactions cycled at 94°C for 5 min of an initial step, followed by 35 cycles of 94°C for 30 s in a denaturation step. The selected temperatures of the annealing step were 42.5–44.1°C for COI, 45–48°C for 16S, and 55–58.1°C for 28S, for 30 s, 72°C for 15 s in an extension step, and then a final extension step at 72°C for 10 min. PCR cycler was installed at a holding temperature at 4°C as the final step.

The PCR products were inspected under 1% (w/v) agarose gel electrophoresis in 0.5x TBE buffer. The fluorescence of PCR bands was enhanced with SYBR Safe illuminant and observed under a UV light source. The gene target products were purified using a QIAquick purification kit (QIAGEN Inc.). The purified PCR products were directly cycle-sequenced using the original amplification primers with an Applied Biosystems automatic sequencer (ABI 3730XL) at Macrogen and Bioneer Inc. (Korea). Sequences were aligned with libraries in GenBank using the BLASTn algorithm to verify the correct group of organisms from product sequences.

### Phylogenetic reconstruction

DNA sequences were assembled in Sequence Navigator [57]. Double strand sequence comparisons were made by a shadow pair-wise alignment function analysis to detect missing sites and gaps in nucleotide sequences, and correlated with chromatograms for each sequence sample. Seven *Scolopendra* species were used in this analysis: *S*. *dawydoffi* Kronmüller, 2012 [30], *S*. *dehaani* Brandt, 1840 [58], *S*. *japonica* Koch, 1878 [59], *S*. *morsitans* Linnaeus, 1758 [60], *S*. *pinguis* Pocock, 1891 [61], *S*. *subspinipes* Leach, 1816 [23] and *Scolopendra* sp. Moreover, sequences of two scolopendromorph taxa from GenBank, *Cormocephalus monteithi* Koch, 1983 [62] and *Cryptops doriae* Pocock, 1891 [61], were chosen as outgroups to root the trees. DNA alignment was carried in MEGA 6 [63] using MUSCLE [64] with the default parameter set. File format preparation, i.e. FASTA, PHYLIP and NEXUS, for further phylogenetic analysis was implemented in MEGA 6 and Mesquite 3.03v [65]. The *heterogeneity of nucleotide substitution* model fit was calculated by JModelTest v.1.7 [66] based on the PhyML likelihood algorithm of heuristic search [67] and MEGA 6. Eleven nucleotide substitution schemes [68] and 88 candidate models were set at the beginning of an analysis for each gene fragment in JModelTest. Gaps and missing data were discarded. For the best fit DNA substitution model, the three fragments were analyzed independently. Kakusan 4 [69], implemented in MOLPHY, was used to assemble the final concatenated file.

In this study, Maximum likelihood (ML) and Bayesian inference (BI) were applied to construct phylogenetic trees from the combination of the partitioned DNA dataset. For ML analysis, the concatenated files were analyzed with Treefinder [70] and RAxML 8.0.0v [71]. A single search was conducted to find the starting tree in Treefinder. Fast likelihood-based analyses were performed with 1,000 bootstrap pseudo-replicates. Bayesian inference was conducted in MrBayes, ver. 3.2.5. [72, 73] using four metropolis-coupled, Markov chain Monte Carlo runs [74]. The program was ordered for random sampling of starting trees before more exhaustive analyses. The number of pseudo-replicates was set at 10,000,000 generations, with simultaneous tree sampling at every 500 random replicates. Sixteen nucleotide substitution schemes and the invisible gamma parameter were applied. Seventy percent of harvested trees were removed as burn-in. The analyses were terminated after the standard deviation of proportional frequency reached below 0.01. The consensus tree implemented from 50% majority rules was obtained at the final stage, and draft tree topology files were reconstructed by FigTree [75]. Node support values have been depicted on the trees in those instances where bootstrap values exceed 70% (ML) and posterior probabilities exceed 0.95 (BI) [74, 76]. A Kimura 2-Parameter model [77] was used to calculate corrected distance of all gene fragments in MEGA 6. Genetic distance was compared for both interspecific and intraspecific variation within and between populations. Finally, species justification and validation of the selected *Scolopendra* species based on genetic affinities are discussed in relation to morphological identification in previous literature.

### Geometric-morphometric analysis

One-hundred seventeen *Scolopendra* specimens were used in this analysis (see S1 Table). Because of morphological changes through the course of ontogeny, a minimum size for sampled individuals was set at 40 mm, following a suggested standard for scolopendrid taxonomy [78, 79]. Three morphological features were examined in this analysis: the cephalic plate, the forcipular coxosternite, and the tergite of the ultimate leg-bearing segment (tergite 21). All samples were photographed in the same orientations and magnifications under a light stereo-microscope. Each feature was analyzed independently by using landmark geometric methods [80, 81]. The landmark points were digitized from a set of stable, conserved parts of each feature, the position detail of each landmark point being as follows:

1. Cephalic plate
  Landmark 1: anterior end of median sulcus of cephalic plate
  Landmark 2: interior basal part of first antennal article (right side)
  Landmark 3: anterior end of anterior ocellus (right side)
  Landmark 4: posterior end of posterior ocellus (right side)
  Landmark 5: intersection point between pleurite of forcipular segment and trochanteroprefemur (right side)
  Landmark 6: intersection point between pleurite of forcipular segment and Tergite 1 (right side)
  Landmark 7: intersection point between pleurite of forcipular segment and Tergite 1 (left side)
  Landmark 8: intersection point between pleurite of forcipular segment and trochanteroprefemur (left side)
  Landmark 9: posterior end of posterior ocellus (left side)
  Landmark 10: anterior end of anterior ocellus (left side)
  Landmark 11: interior basal part of first antennal article (left side)
2. Forcipular coxosternite
  Landmark 1: median diastema
  Landmark 2: inner end of oblique suture (left tooth-plate)
  Landmark 3: outer end of oblique suture (left tooth-plate)
  Landmark 4: left upper corner of forcipular coxosternite
  Landmark 5: coxosternal condyle (left side)
  Landmark 6: inner end of coxosternite collar (left side)
  Landmark 7: left junction between presternite and sternite of first leg-bearing segment
  Landmark 8: right junction between presternite and sternite of first leg-bearing segment
  Landmark 9: inner end of coxosternite collar (right side)
  Landmark 10: coxosternal condyle (right side)
  Landmark 11: right upper corner of coxosternite
  Landmark 12: outer end of oblique suture (right tooth-plate)
  Landmark 13: inner end of oblique suture (right tooth-plate)
3. Tergite of ultimate leg-bearing segment
  Landmark 1: anterior interior margin (right side)
  Landmark 2: anterior exterior margin (right side)
  Landmark 3: posterior exterior margin (right side)
  Landmark 4: posterior exterior margin (right side)
  Landmark 5: distal point of postero-median margin
  Landmark 6: posterior exterior margin (left side)
  Landmark 7: posterior exterior margin (left side)
  Landmark 8: lower exterior margin (left side)
  Landmark 9: upper interior margin (left side)

All landmark points were marked manually by WS with tpSDig2 [82]. The standard image of each constant character of all samples was randomly chosen with tpsUtil [83] in order to avoid personal bias. MorphoJ 1.06b [84] was used for testing shape variation. Procrustes superimposition was calculated to minimize effects such as sample size, orientation and depth [85, 86]. The covariance metric was generated as two-dimensional axes for each feature. Multivariate regression was performed using the Procrustes superimposed data to define allometry and statistically test for correlation between centroid origin and shape variation [87]. A category of sampled specimens was classified based on morphological identification. Canonical variates analysis (CVA) implemented the relative determination of two or more classified groups under Mahalanobis and Procrustes distance values [88, 89]. A permutation test for pairwise distance was set at 10,000 permutation rounds for calculation of Mahalanobis distance in both between- and among-classified groups. From the CVA results, the shape variation detected from landmark positions was linked serially as Wire-frame outlines to visualize shape reformation between negative and positive canonical variates groups on a three-dimensional axis. Confidence ellipses were calculated to indicate the centroid origin of each defined sample group in a three-dimensional CVA graph. Comparative CVA plots were generated separately in each dimension for CV1-CV2 and CV2-CV3. All graphs were exported in Encapsulated Postscript Vector Graphics format (EPS) for processing in Adobe Illustrator.

## Results

### Morphological identification

This study is based on 176 centipede specimens collected from 134 localities in mainland Southeast Asia and one voucher specimen from the Japanese archipelago. All specimens were observed by light microscopy, with taxonomy based on traditional external morphological characters of scolopendrids. These are as follow: the number of antennal articles, as well as the number of those that are sparsely hirsute (“glabrous”); number of teeth on the forcipular coxosternal tooth-plates; the first tergite to possess complete paramedian sutures; the first tergite with complete margination; the extent of paramedian sutures on the sternites (complete or confined anteriorly to a variable extent); the number of spines on the coxopleuron (specifically, the number of apical spines and the presence/numbers of subapical and dorsal spines); the prefemoral spine arrangement on the ultimate legs; the presence or absence of tarsal spurs on legs 19 and 20; and, presence or absence of a gonopod (“genital appendage”) on the first genital segment of the male. The taxonomic results show six nominal species that can be identified as named species and one putative new species in the sampling area as follow: *Scolopendra dawydoffi*, *S*. *dehaani*, *S*. *japonica*, *S*. *morsitans*, *S*. *subspinipes* and *Scolopendra* sp. Diagnostic character combinations of all assigned species are summarized in Table 1.

**Table 1:** Diagnostic description of all examined species based on external morphology and common colouration schemes of voucher specimens in this analysis, with references to recent taxonomic descriptions with additional information.

		Colouration pattern	
Taxon and recent taxonomic references	Diagnostic description/Type locality/ distribution	Immature stage	Mature stage
*S*. *dawydoffi* [30]	17–18 antennal articles, 6 basal articles glabrous dorsally. 5–10 teeth on tooth plate. Tergites with paramedian sutures starting from TT2-3. Complete tergite margination from TT12(14). Paramedian sutures on anterior 20–60% of sternites. Coxopleuron with 2–3 apical spines. Ultimate legs with 2 VL, 1 M, 1–2 DM and 1–3 corner spines on prefemur. Tarsal spur on legs 1–19. Male gonopods absent. **Type locality:** Laos; Thakek, Vietnam: Hagiang, Haut Tonkin. **Distribution:** Laos, Vietnam, Cambodia and Thailand	n/a	D: Cephalic plate and anterior part of tergites reddish; posterior part of tergites with transverse blackish band
*S*. *dehaani* [30, 119]	18–21 antennal articles, 5 basal articles glabrous dorsally. 5 teeth on tooth plate. Tergites with paramedian sutures starting from TT3-4. Complete tergite margination from T7. Complete paramedian sutures on sternites. Coxopleuron with 2 apical spines. Ultimate legs with 0–1 M, 0–1 DM and 3 corner spines on prefemur. Tarsal spur on legs 1–20. Male gonopods present. **Type locality:** Java, Indonesia. **Distribution:** SE-Asian countries, Japan, India and Bangladesh	D: Cephalic plate greenish blue; tergites yellow with dark band on posterior part	D: Cephalic plate reddish brown; tergites entirely black. M: Entirely black or reddish brown on all segments
*S*. *japonica* [30, 37]	16–18 antennal articles, 6 basal articles glabrous dorsally. 5–6 teeth on tooth plate. Tergites with paramedian sutures starting from T4. Complete tergite margination from T12. Complete paramedian sutures on sternites. Coxopleuron with 3 apical spines. Ultimate legs with 2–3 VL, 1 M, 2 DM and 3–4 corner spines on prefemur. Tarsal spur on legs 1–19. Male gonopods present. **Type locality:** Japan. **Distribution:** Japan	n/a	D: Cephalic plate yellowish or brown; tergites greenish
*S*. *pinguis* [22, 61]	17 antennal articles, 4 basal articles glabrous dorsally. 6 teeth on tooth plate. Tergites with paramedian sutures starting from T3. Complete tergite margination from T16(18). Paramedian sutures on anterior 10–30% of sternites. Coxopleuron with 3–7 apical, 1 subapical 1–2 lateral and 2–3 dorsal spines. Ultimate legs with 2–9 VL, 0–6 VM, 2–3 M, 2 DM and 1–2 corner spines on prefemur. Tarsal spur present on legs 1-19(21). Male gonopods present. **Type locality:** Cheba Dist., Carin mountains, Myanmar (Burma). **Distribution:** Myanmar, Thailand and Laos	D: Cephalic plate yellowish; tergites black. M: Entirely black	D: Cephalic plate yellowish; tergites black. M: Entirely black on all segments
*S*. *morsitans* [37, 157]	18–19 antennal articles, 6 basal articles glabrous dorsally. 6 teeth on tooth plate. Tergites with paramedian sutures starting from T7(12), incomplete on anterior and posterior part. Complete paramedian sutures on sternites. Coxopleuron with 2–3 apical spines and 1 dorsal spine. Ultimate legs with 2 VL, 0–1 M, 0–1 DM and 2 corner spines on prefemur. Tarsal spur present on legs 1-19(20). Male gonopods present. **Type locality:** India. **Distribution:** Worldwide	D: Cephalic plate reddish; tergites brown with dark band on median part	D: Cephalic plate reddish; tergites brownish with transverse pigmented band on posterior part
*S*. *subspinipes* [30]	19 antennal articles, 6 basal articles glabrous dorsally. 7 teeth on tooth plate. Tergites with paramedian sutures starting from T3. Complete tergite margination from T14(17). Complete paramedian sutures on sternites. Coxopleuron with 2 apical spines. Ultimate legs with 2 VL, 1 M, 1 DM and 2 corner spines on prefemur. Tarsal spur on legs 1–20. Male gonopods present. **Type locality:** Not designated. **Distribution:** Worldwide	D: Cephalic plate greenish blue; tergites yellow with dark band on posterior part	D: Cephalic plate reddish; tergites brownish or black. M: Reddish or brown on all segments
*Scolopendra* sp.	18–19 antennal articles, 6 basal articles glabrous dorsally. 6 teeth on tooth plate. Tergites with paramedian sutures starting from T7 (12). Tergites with incomplete paramedian sutures on anterior and posterior part. Paramedian sutures on anterior 15–20% of sternites. Coxopleuron with 2–3 apical spines and 1 dorsal spine. Ultimate legs with 2 VL, 0–1 M, 0–1 DM and 2 corner spines on prefemur. Tarsal spur on legs 1-19(20). **Type locality:** Not designated. **Distribution:** Laos	n/a	M: Entirely black or greenish black on all segments

*D- Dichromatic pattern and M- monochromatic pattern

### Sequence annotation

Sixty nucleotide sequences from partial gene targets for cytochrome *c* oxidase subunit 1, 16S rDNA and 28S rDNA were obtained (Table 2). All raw nucleotide sequences were blasted with other available scolopendromorph sequences in GenBank as a check for contamination. The compatibility values of all sequence reached up to 80% of available scolopendromorph sequences, suggesting that outgroup contamination is not affecting the genomic DNA. The final aligned sequences obtained from sequence editing and the alignment program, consisted of 814 bp for COI, 446 bp for 16S, and 638 bp for 28S. Sequence annotation (Table 3) of each gene is as follows: COI sequences consist of 334 parsimony-informative sites, and 403 and 411 variable and conservative sites, respectively; 16S sequences comprise 197 parsimony-informative sites, and 271 and 175 variable and conservative sites, respectively; 28S sequences include 110 parsimony-informative sites, and 206 and 432 variable and conservative sites, respectively. Corrected genetic distances were calculated under the Kimura-2-parameter model for DNA sequence alignment. Interspecific variation in each partial sampling of genes is 15–24.2% for COI, 10.6–22.4% for 16S, and 0.8–10.8% for 28S. A summary of inter-intra specific variation and best fit scores for the nucleotide substitution model is given in Tables 4 and 5.

**Table 2:** List of voucher specimens of seven *Scolopendra* species and selected outgroups used in phylogenetic analyses. Each sample includes the collecting locality, GPS co-ordinates, CUMZ registration numbers, and GenBank accession number for three selected genes (COI, 16S and 28S).

				GenBank accession Nos		
Species/Locality	GPS coordinates	Sample names	CUMZ Nos.	COI	16S	28S
*Scolopendra dawydoffi* Kronmüller, 2012						
**1. Saphan Hin Waterfall, Trad**	12°06’07.7”N 102°42’38.8”E	E6	00272	KR705680	KR705618	KR705742
**2. Sakearat, Nakhon Ratchasima**	14°30’36.5”N 101°55’51.5”E	NE12	00290	KR705654	KR705592	KR705716
**3. Wat Thang Biang, Pak Chong, Nakhon Ratchasima**	14°32’22.0”N 101°21’54.6”E	Sub 4, Sub 5	00294.1–2	KR705635, KR705634	KR705573, KR705572	KR705697, KR705696
*Scolopendra dehaani* Brandt, 1840						
**4. Wang Kanlueang Waterfall, Lopburi**	15°06’49.4”N 101°06’38.8”E	C5	00282	KR705689	KR705627	KR705751
**5. Sapanthai, Bangban, Ayutthaya**	14°21’51.4”N 100°29’22.3”E	C6	00256	KR705688	KR705626	KR705750
**6. Lan Island, Rayong**	12°55’05.8”N 100°46’43.8”E	E1	00320	KR705684	KR705622	KR705746
**7. Wat Khao Chakan, Srakaeo**	13°39’32.3”N 102°05’10.7”E	E4	00321	KR705682	KR705620	KR705744
**8. Tha Sen Waterfall, Trad**	12°07’59.1”N 102°42’22.6”E	E5	00322	KR705681	KR705619	KR705743
**9. Sichang Island, Chonburi**	13°09’04.7”N 100°48’55.6”E	E16	00252	KR705683	KR705621	KR705745
**10. Wat Tham Chiangdao, Chiangmai**	19°23’36.8”N 98°55’42.6”E	N3	00323	KR705659	KR705597	KR705721
**11. Hui Hong Khrai, Chiangmai**	18°50’58.6”N 99°13’18.9”E	N4	00346	KR705658	KR705596	KR705720
**12. Wat Ban Mai, Maehongson**	19°17’55.3”N 97°59’13.5”E	N6	00324	KR705657	KR705595	KR705719
**13. Pha Mon Cave, Pangmapha, Maehongson**	19°30’01.6”N 98°16’43.5”E	N7	00325	KR705656	KR705594	KR705718
**14. Ban Dongsavanh, Phang Khon, Sakon Nakhon**	16°50’59.5”N 103°22’40.4”E	NE1	00247	KR705655	KR705593	KR705717
**15. Ban Thatoom, Mahasarakarm**	16°10’32.2”N 103°26’59.6”E	NE2	00275	KR705651	KR705589	KR705713
**16. Wat Tham Phapu, Loei**	17°34’41.5”N 101°42’39.1”E	NE14	00277	KR705653	KR705591	KR705715
**17. Kaeng Lamduan, Namyeun, Ubon Ratchathani**	14°26’15.0”N 105°06’06.7”E	NE15	00248	KR705652	KR705590	KR705714
**18. Wang Thong Cave, Khuon Khanun, Phatthalung**	7°40’55.1”N 100°00’56.8”E	S1	00274	KR705641	KR705579	KR705703
**19. Klong Phot Waterfall, Nopphitam, Nakon Si Thammarat**	7°48’37.8”N 99°12’20.0”E	S3	00281	KR705639	KR705577	KR705701
**20. JPR Stone Park, Kraburi, Ranong**	10°29’36.7”N 98°54’35.7”E	S5	00262	KR705637	KR705575	KR705699
**21. Sairung Waterfall, Takua Pa, Phangnga**	8°44’30.2”N 98°16’45.4”E	S27	00251	KR705640	KR705578	KR705702
**22. Kreab Cave, Langsuan, Chumporn**	9°49’01.8”N 99°02’15.6”E	S31	00326	KR705638	KR705576	KR705700
**23. Hub Pa-Tat, Lansak, Uthaithani**	15°22’37.4”N 99°37’51.9”E	W1	00243	KR705632	KR705570	KR705694
**24. Khao Marong, Prachuap Khirikhan**	11°12’22.0”N 99°29’45.8”E	W3	00327	KR705628	KR705566	KR705690
**25. Wat Tham Lijia, Sangkhlaburi, Kanchanaburi**	15°04’12.8”N 98°33’56.4”E	W10	00328	KR705631	KR705569	KR705693
**26. Tham Khao Bin, Ratchaburi**	13°35’35.6”N 99°40’02.3”E	W12	00253	KR705630	KR705568	KR705692
**27. Wat Phothikhun, Maesot, Tak**	16°44’39.2”N 98°36’17.2”E	W17	00329	KR705629	KR705567	KR705691
**28. Angkor Wat, Siem Reap, Cambodia**	13°24’45.5”N 103°52’14.7”E	CM1	00330	KR705687	KR705625	KR705749
**29. Wat Tham Ban Kele, Srisophon, Cambodia**	13°36’05.5”N 102°57’09.3”E	CM2	00331	KR705686	KR705624	KR705748
**30. Hin Tang Stone field, 39 Km. before Vietnam border, Attapu, Laos**	14°43’18.6”N 107°17’39.6”E	L1	00332	KR705678	KR705616	KR705740
**31. Khon Phapaeng Waterfall, Champasak, Laos**	13°56’53.2”N 105°56’27.1”E	L2	00333	KR705673	KR705611	KR705735
**32. Luang Prabang, Laos**	19°53’10.2”N 102°08’16.2”E	L11	00334	KR705677	KR705615	KR705739
**33. Ban Bun-Tai, Bun-Tai, Phongsali, Laos**	21°26’50.8”N 101°58’30.5”E	L14	00335	KR705676	KR705614	KR705738
**34. Gumpung Baru, Gunung Getting, Perak, Malaysia**	4°41’39.9”N 100°52’46.0”E	Ma1	00336	KR705669	KR705607	KR705731
**35. Gua Musang, Kelantan, Malaysia**	4°52’11.3”N 102°00’40.6”E	Ma2	00337	KR705668	KR705606	KR705730
**36. Klinic Desa, Kampung Panit Luar, Perak, Malaysia**	4°56’17.9”N 100°59’00.1”E	Ma3	00338	KR705667	KR705605	KR705729
*Scolopendra japonica* Koch, 1860						
**37. Shinshu University, Matsumoto, Japan**	36°13’22.4”N 137°54’35.0”E	JP1	00319	KR705679	KR705617	KR705741
**38. Plain of Jar, Xieang Khouang, Laos**	19°25’51.5”N 103°09’10.4”E	L7, L8	00298.1–2	KR705671, KR705670	KR705609, KR705608	KR705733, KR705732
**39. Phu Fah Mountain, Phongsali, Laos**	21°41’19.6”N 102°06’30.4”E	L16, L17	00297.1–2	KR705675, KR705674	KR705613, KR705612	KR705737, KR705736
*Scolopendra morsitans* Linnaeus, 1758						
**40. Ban Dan Chang, Ta Kantho, Khonkaen**	16°50’06.1”N 103°16’32.0”E	MS5	00339	KR705662	KR705600	KR705724
**41. Lainan, Weing Sa, Nan**	18°34’16.1”N 100°46’59.7”E	MS6	00340	KR705661	KR705599	KR705723
**42. Juang Island, Sattahip, Chonburi**	12°31’46.4”N 100°57’18.4”E	MS7	00341	KR705660	KR705598	KR705722
**43. Ban Khok Pho, Prasat, Surin**	14°32’53.4”N 103°22’19.1”	MS11	00342	KR705666	KR705604	KR705728
**44. Hui Hong Khrai, Chiangmai**	18°50’59.5”N 99°13’16.4”E	MS12	00343	KR705665	KR705603	KR705727
**45. Tha Kra Bak Reservoir, Srakaeo**	13°58’13.9”N 102°15’57.6”E	MS14	00344	KR705664	KR705602	KR705726
**46. Wat Phanombak, Srisophon, Cambodia**	13°36’05.5”N 102°57’09.3”E	MS18	00345	KR705663	KR705601	KR705725
*Scolopendra pinguis* Pocock, 1891						
**47. Chong Kao Khat, Kanchanaburi**	14°22’47.6”N 98°55’47.7”E	P1	00312	KR705650	KR705588	KR705712
**48. Phusang Waterfall, Phayao**	19°37’10.2”N 100°21’54.7”E	P2	00305	KR705647	KR705585	KR705709
**49. Khao Rao Cave, Bokaeo, Laos**	20°41’56.6”N 101°05’46.8”E	P3	00309	KR705646	KR705584	KR705708
**50. Wat Tham Lijia, Sangkhlaburi, Kanchanaburi**	15°04’12.8”N 98°33’56.4”E	P4	00303	KR705646	KR705584	KR705708
**51. Hui Nam-Un, Wiangkhum, Nan**	18°30’22.8”N 100°31’49.1”E	P5	00307	KR705644	KR705582	KR705706
**52. Ban Na-Ton, Muang Khun, Xieng Khouang, Laos**	17°52’31.4”N 104°51’44.7”E	P6	00306	KR705643	KR705581	KR705705
**53. Wiang Thong Hotspring, Mueang Ieam, Huaphan, Laos**	20°04’45.2”N 103°44’33.3”E	P7	00304	KR705642	KR705580	KR705704
**54. Ban Pang Pan, Maetaeng, Chiangmai**	19°12’17.4”N 98°40’00.7”E	P11	00313	KR705649	KR705587	KR705711
**55. Phamone Cave, Pangmapha, Maehongson**	19°30’01.6”N 98°16’43.5”E	P13	00314	KR705648	KR705586	KR705710
*Scolopendra subspinipes* Leach, 1814						
**56. Kentridge Rd., Singapore**	1°17’08.9”N 103°47’09.8”E	SP1	00315	KR705636	KR705574	KR705698
*Scolopendra* sp.						
**57. Tat E-tu, Pakse, Champasak, Laos**	15°13’10.6”N 105°55’31.3”E	L6	00316	KR705672	KR705610	KR705734
**58. Tat Pha Yueang, Mueang Sing, Luang Namtha, Laos**	15°09’55.1”N 106°06’10.6”E	U	00317	KR705633	KR705571	KR705695
*Cormocephalus monteithi* Koch, 1983						
**59. n/a**	n/a	outgroup	n/a	HM453309.1*	AF370861.1*	HM453274*
*Cryptops doriae* Pocock, 1891						
**60. Doi Inthanon, Chom Thong, Chiangmai**	18°35'17.9"N 98°29'09.5"E	outgroup	00318	KR705685	KR705623	KR705747

* sequence from Murienne et al. (2011) [109].

**Table 3:** Characteristics of nucleotide sequence for three amplified genes and best fit models of heterogeneous nucleotide substitution for each gene calculated from jModel Test under AIC and BIC criteria.

	Sequence analysis				Nucleotide substitution model test			
	Sequence length	Informative sites	Variable sites	Conservative sites	Fit model for ML	AIC	Fit model for BI	BIC
COI	814	334	403	411	JC	27387.768	JC	27970.811
16S	446	197	271	175	GTR+G	10228.203	GTR+G+I	107778.6242
28S	638	110	206	432	GTR+G+I	5021.643	GT+G	5614.864

**Table 4:** Corrected distance of interspecific variation in seven *Scolopendra* species from COI and 16S partial gene analyses under calculation model of K-2 parameter.

		COI						
	Taxon	*Scolopendra dehaani*	*Scolopendra* sp.	*Scolopendra dawydoffi*	*Scolopendra subspinipes*	*Scolopendra pinguis*	*Scolopendra morsitans*	*Scolopendra japonica*
**16S**	***Scolopendra dehaani***		0.150	0.165	0.170	0.209	0.209	0.194
	***Scolopendra* sp.**	0.106		0.182	0.199	0.217	0.220	0.201
	***Scolopendra dawydoffi***	0.130	0.134		0.188	0.209	0.200	0.166
	***Scolopendra subspinipes***	0.129	0.137	0.137		0.219	0.242	0.220
	***Scolopendra pinguis***	0.201	0.217	0.194	0.208		0.238	0.207
	***Scolopendra morsitans***	0.187	0.196	0.209	0.189	0.224		0.227
	***Scolopendra japonica***	0.128	0.101	0.108	0.134	0.224	0.193	

**Table 5:** Corrected distance of intraspecific variation in six *Scolopendra* species from COI, 16S and 28S partial gene analysis under calculation model of K-2 parameter.

Taxon	COI	16S	28S
***Scolopendra dehaani***	0.086	0.047	0.001
***Scolopendra* sp.**	0.122	0.15	0.003
***Scolopendra dawydoffi***	0.02	0.009	0
***Scolopendra subspinipes***	n/c	n/c	n/c
***Scolopendra pinguis***	0.183	0.111	0.01
***Scolopendra morsitans***	0.086	0.063	0.006
***Scolopendra japonica***	0.124	0.053	0.003

### Phylogenetic analysis

**Fig 1:**
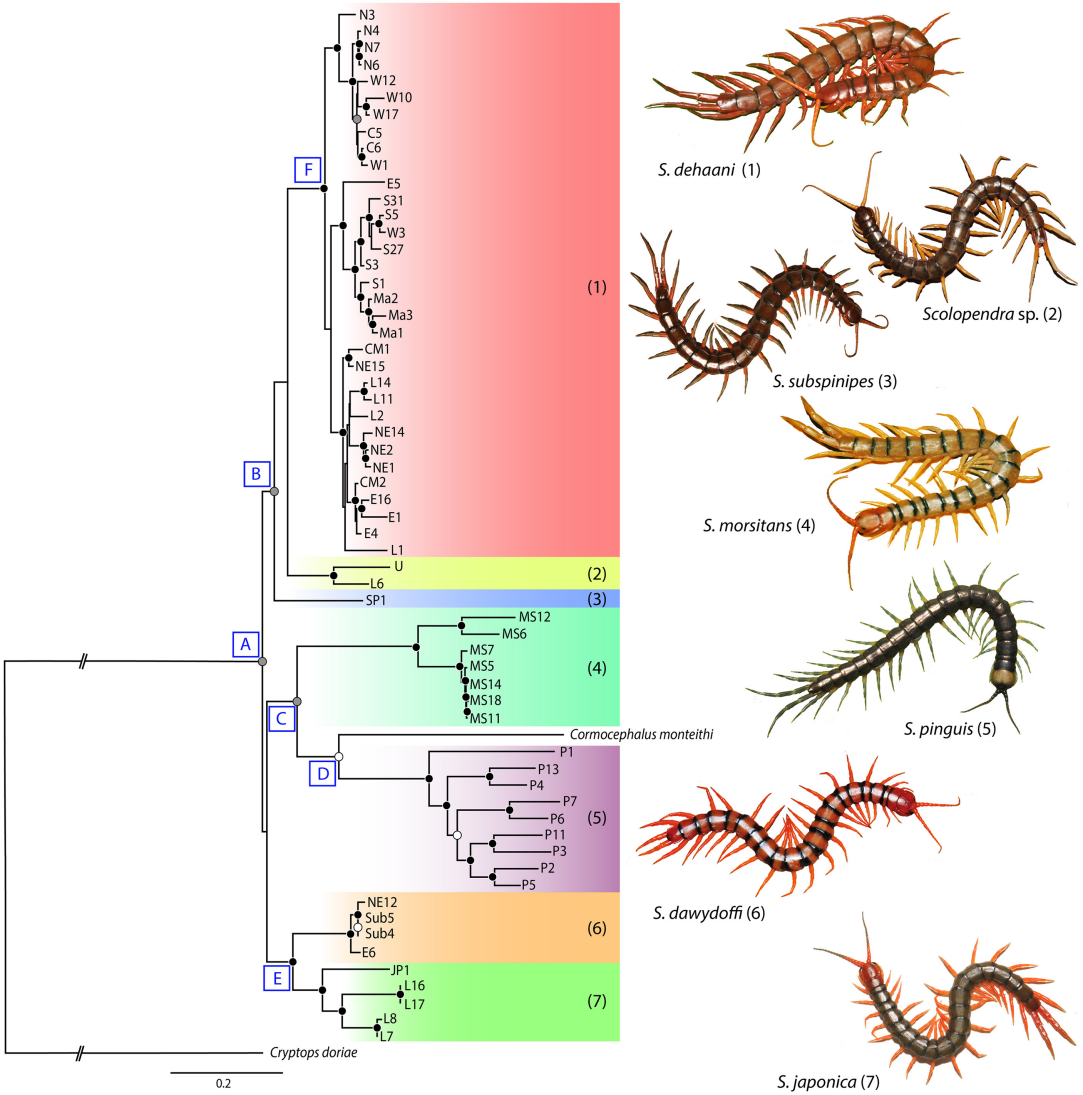
Phylogenetic tree of *Scolopendra* mainland Southeast Asia. Relationships among *Scolopendra* and two outgroups indicated similarly both in Maximum likelihood (ML) and Bayesian inference (BI) of the concatenated COI, 16S and COI partial gene analyses. Significant support values in ML and BI are indicated by three colouration circles; black circle = support both in ML and BI (above 70% bootstrap in ML and 0.95 posterior probability in BI), white circle = support only in ML, grey circle = support only in BI. The gradient colouration bars on the tree represent the genetic affinities of populations relative to morphological identification in each species.

The best fit models of heterogeneous nucleotide substitution under the two optimality criteria (Maximum likelihood and Bayesian inference) for each partial gene analysis are JC, GTR+G+I and GTR+G (Table 3). The output trees for the concatenated analyses have congruent topologies in both analyses (Fig 1; see S1 Fig for node support). They depict the expected monophyly of the subfamily Scolopendrinae Kraepelin, 1903 [90], with all species of *Scolopendra* and the outgroup OTU of *Cormocephalus monteithi* nesting together with strong posterior probability support in BI (Fig 1A). Inside the Scolopendrinae, however, the monophyly of the genus *Scolopendra* is contradicted by the interpolation of *C*. *monteithi* within it (Fig 1D). In the case of examined *Scolopendr*a taxa, seven genetically delimited taxa can be discriminated that are congruent with their morphological identification as species, and phylogeographic structure is resolved within each of these species. The tree separates members of *Scolopendra* into three main clades. One clade includes *S*. *dehaani*, a putatively new species that we refer to as *Scolopendra* sp., and *S*. *subspinipes* (labeled as clade B in Fig 1), and another unites *S*. *morsitans* and *S*. *pinguis* (clade C in Fig 1). The third clade (clade E in Fig 1) groups *S*. *japonica* and *S*. *dawydoffi*. From the tree topology, short internal branch lengths are characteristic of populations within *S*. *dehaani* (Fig 1, clade F therein) while the remaining species showed greater amounts of genetic diversity between their populations.

**Fig 2:**
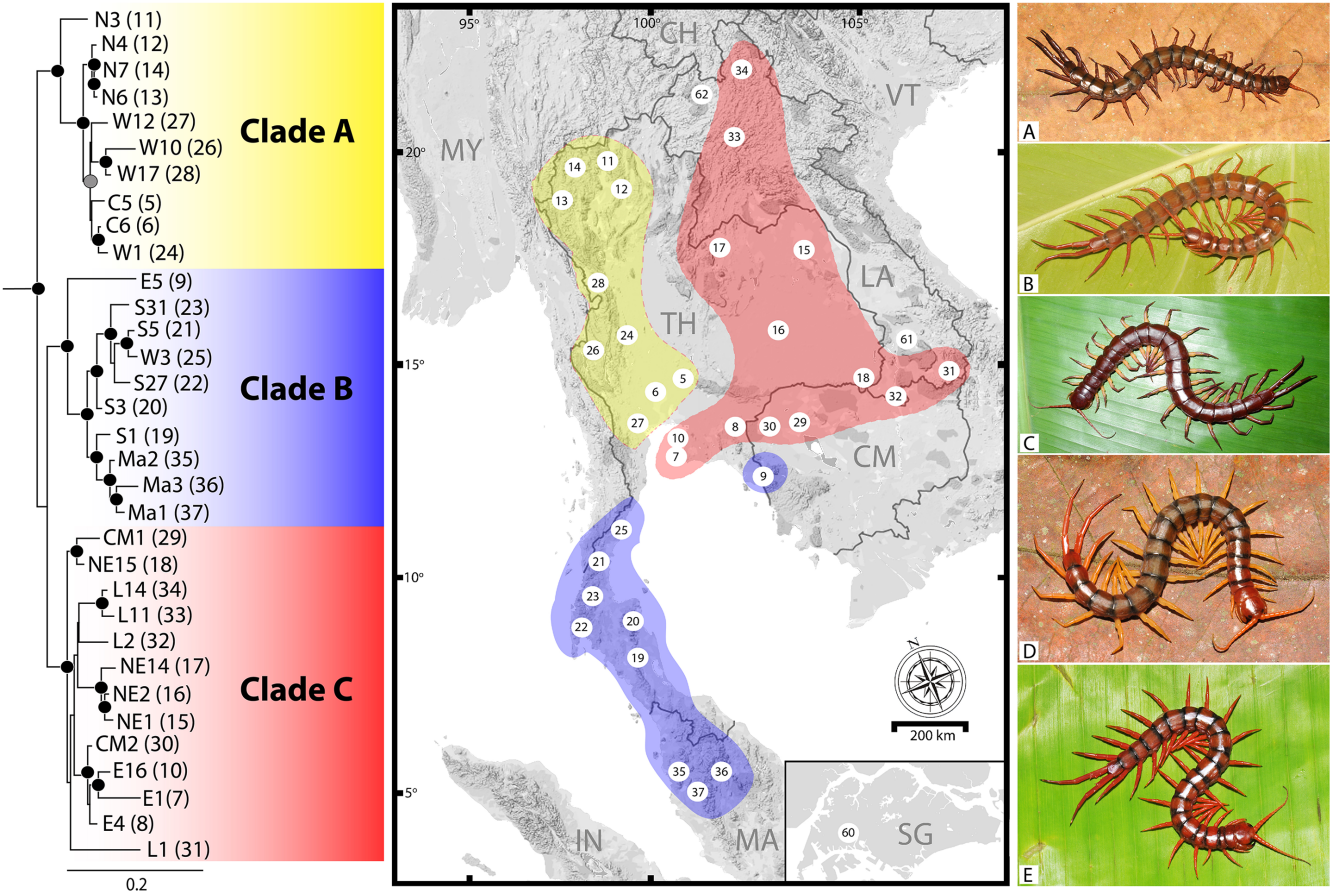
Phylogenetic relationship of *S*. *dehaani* population (left) based on genetic structure among its populations relative to regional distribution in mainland Southeast Asia (middle); colours indicate the major populations. Five patterns of live colour morphs in *S*. *dehaani* were found (right); A. Dark colour morph; B. Light brownish colour morph; C. Reddish-brown body color morph, yellowish legs with reddish on distal part; D. Dichromatic pattern; cephalic plate, tergite 1, 20 and 21 reddish, tergites 2–19 brownish with yellowish legs; E. Reddish colour morph with dark band on anterior and posterior parts of tergites.

In *S*. *dehaani* (Fig 2), the phylogenetic tree indicates three major groups that have clear relationships to geographical zones as follow: the Chao Phraya Basin population (CPB; Clade A), the Mekong River Basin population (MRB; Clade C), and the Lower Tenasserim Range population (LTR; Clade B). The CPB clade unites populations from the northern, western (upper part of the Tenasserim Range) and central parts of Thailand. The northern population is separated from the others with strong node support in ML and BI analysis (73/1) while the relationship between MRB and LTR remains unresolved, with weak support values in ML and BI (64/0.86). The MRB clade consists of all populations from northeastern and eastern parts of Thailand, Laos and some western Cambodian populations. Inside the MRB clade, node support values indicate that the western Cambodian population and the lower northeastern Thailand population are closely related (98/1; ML and BI) while relationships in the other populations are undefined. The LTR clade includes southern populations starting from the Isthmus of Kra through the coastal part of Thailand to the northern part of the Malay Peninsula. In this clade, the tree topology indicates two main populations, one northern and other southern. The split between these two clades received strong support both in ML and BI (98/1). In addition, genetic relationships are congruent with colouration among regional populations of *S*. *dehaani* in the Southeast Asian mainland. During field surveys, five colouration patterns have been recorded (Fig 2A–2E). Colour morphs 2–4 are found throughout most regions whereas morphs 1 and 5 (Fig 2A and 2E) are specific to only some populations. Colour morph 1 (Fig 2A) has been reported only from some specimens of the CPB population. This colour morph has a dark brown body and dark violet legs, with most specimens coming from the north western part of Thailand, close to the Thailand-Myanmar border. Also geographically restricted is colour morph 5 (Fig 2E), which has been found only in the LTR population. In this, the body is bright reddish with a dark band on the posterior part of the tergites while all legs are usually reddish or yellowish.

*Scolopendra* sp. and *S*. *subspinipes* are the closest relatives of *S*. *dehaani* (Fig 1; clade B), although the precise sister group relationships are equivocal because the putative clade that unites *Scolopendra* sp. and *S*. *dehaani* to the exclusion of *S*. *subspinipes* has low support values in both ML and BI (39/0.63). The union of the two sampled populations of *Scolopendra* sp. from northern and southern Laos supports the validity of this species, as is indicated by diagnostic morphological characters, notably the incomplete paramedian sutures on the tergites. In case of *S*. *subspinipes*, the tree topology indicated the separation of *S*. *subspinipes* from *S*. *dehaani* as well as *Scolopendra* sp. according to both support values (56/0.97) and corrected distances (12.9–13.7% and 17–19.9% in COI and 16S, respectively).

**Fig 3:**
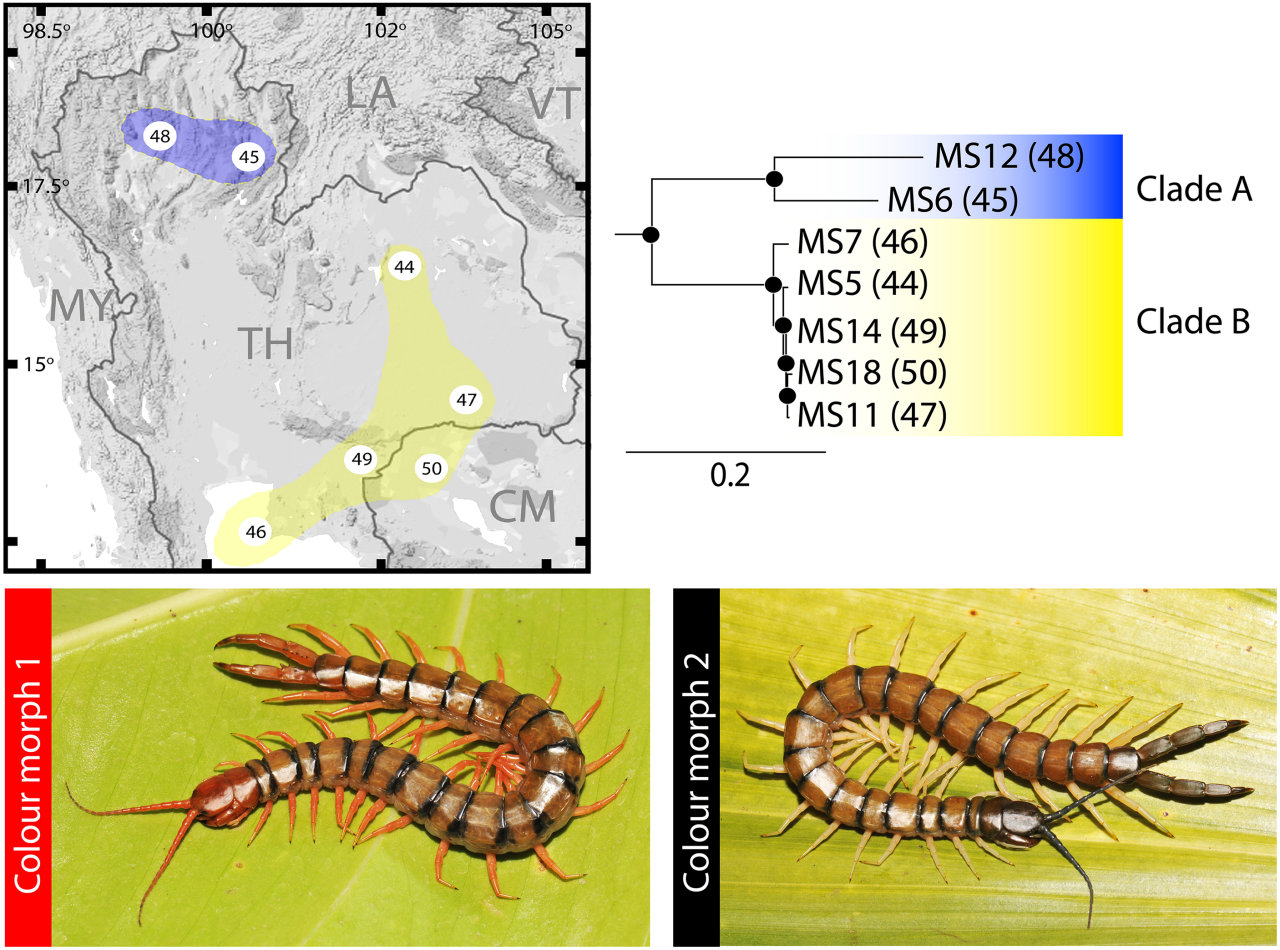
Phylogenetic tree of *S*. *morsitans*. Colour gradient indicates population structure; blue gradient indicates the northern population, yellow gradient the eastern population. *Scolopendra morsitans* exhibited two colour morphs: colour morph 1—antenna, cephalic plate, tergites 1, 20 and 21 and ultimate legs orange; colour morph 2—antenna, cephalic plate, tergites 1, 20 and 21 and ultimate legs blackish.

*S*. *morsitans* was nested in the same clade with *S*. *pinguis* and an outgroup (*C*. *monteithi*) (Fig 1; clade C). All *S*. *morsitans* populations were nested together as monophyletic. However, the genetic affinity divided *S*. *morsitans* into two minor populations (Fig 3), one of which is found in the northern part of Thailand (clade A in Fig 3) whereas the other is located in northeastern Thailand and some populations from Cambodia. This separation was supported with strong nodal support in both ML and BI (100/1) whether between or within the two populations. Furthermore, observation of colour patterns indicated two colour morphs for *S*. *morsitans* in this region (Fig 3). The most common pattern, called colour morph 1, covers most populations in this region. The cephalic plate, tergite 21 and ultimate legs of this morph are red-yellowish, while colour morph 2 has the cephalic plate, tergite 21 and ultimate leg blackish. This latter morph is only found in the north-western population.

**Fig 4:**
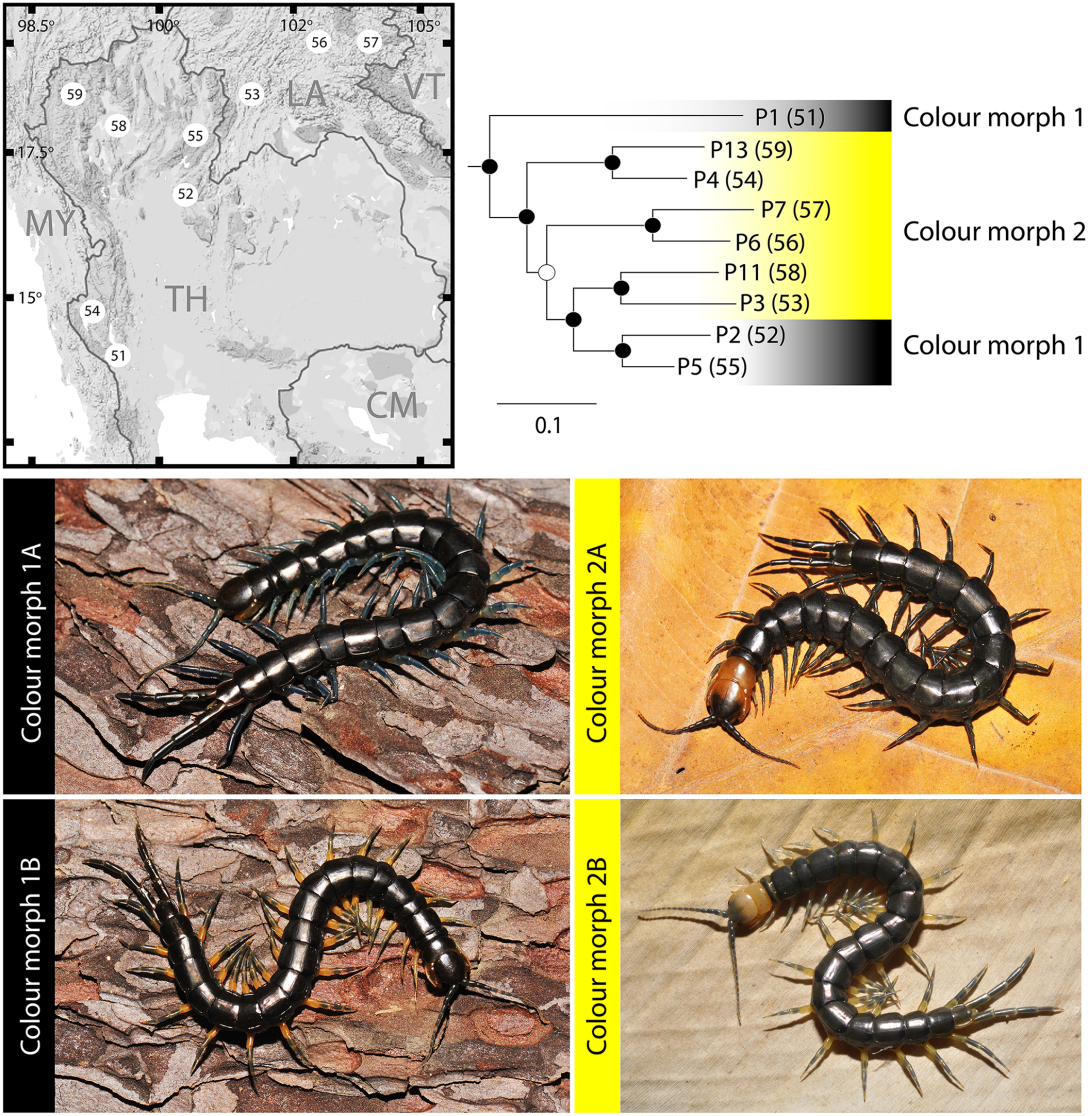
Phylogenetic tree of *S*. *pinguis* based on genetic structure of its populations. Colour gradient bar indicates colour morphs of sampled individuals that divide into two patterns; colour morp 1—blackish population (monochromatic); colour morph 2- yellowish—black population (dichromatic). Four live colour morph pictures depict the variability of colouration on legs of the two colour morphs in *S*. *pinguis*; colour morph 1A and 2A—animal with dark blue legs, colour morph 2A and 2B—animal with yellowish legs with blue stripes on distal part.

The tree shows marked genetic diversity between populations of *S*. *pinguis*, as indicated both by branch lengths and the corrected within-species distances (18% and 11% in COI and 16S, respectively). Monophyly of the species is supported (Fig 4), as is consistent with its diagnostic morphological characters, and groupings within the species received strong support in both ML and BI analysis. The genetic structure of *S*. *pinguis* showed that Thai populations exhibited a genetic connection with all examined populations from Laos. Moreover, the tree topology depicts a relationship between western and northwestern Thailand, which showed closer affinities with each other than with other populations. However, one population (Fig 4; P1) from the western part of Thailand did not group with the adjacent population (Fig 4; P4) and the former is resolved basally relative to all other *S*. *pinguis* populations. This species also exhibited colour variation among its populations, as for the previously discussed species. Four colouration patterns (Fig 4) have been recorded both in the Thai and Laotian faunas. The major colouration pattern is seen on the cephalic plate, which differs between yellowish and blackish colour morphs. Minor variability has also been detected on the legs, these exhibiting two colour forms, either being monochrome or dichromatic. The tree topology indicated that all populations with the yellowish cephalic plate were grouped together but one of the two blackish populations is nested within the yellowish population. The blackish populations are divided into two lineages, one of which is sister lineage to a clade that unites all other sampled individuals. With regards to their geographical distribution, the blackish cephalic plates are specific to the north western area of Thailand whereas the other forms are distributed both in Thailand and Laos.

**Fig 5:**
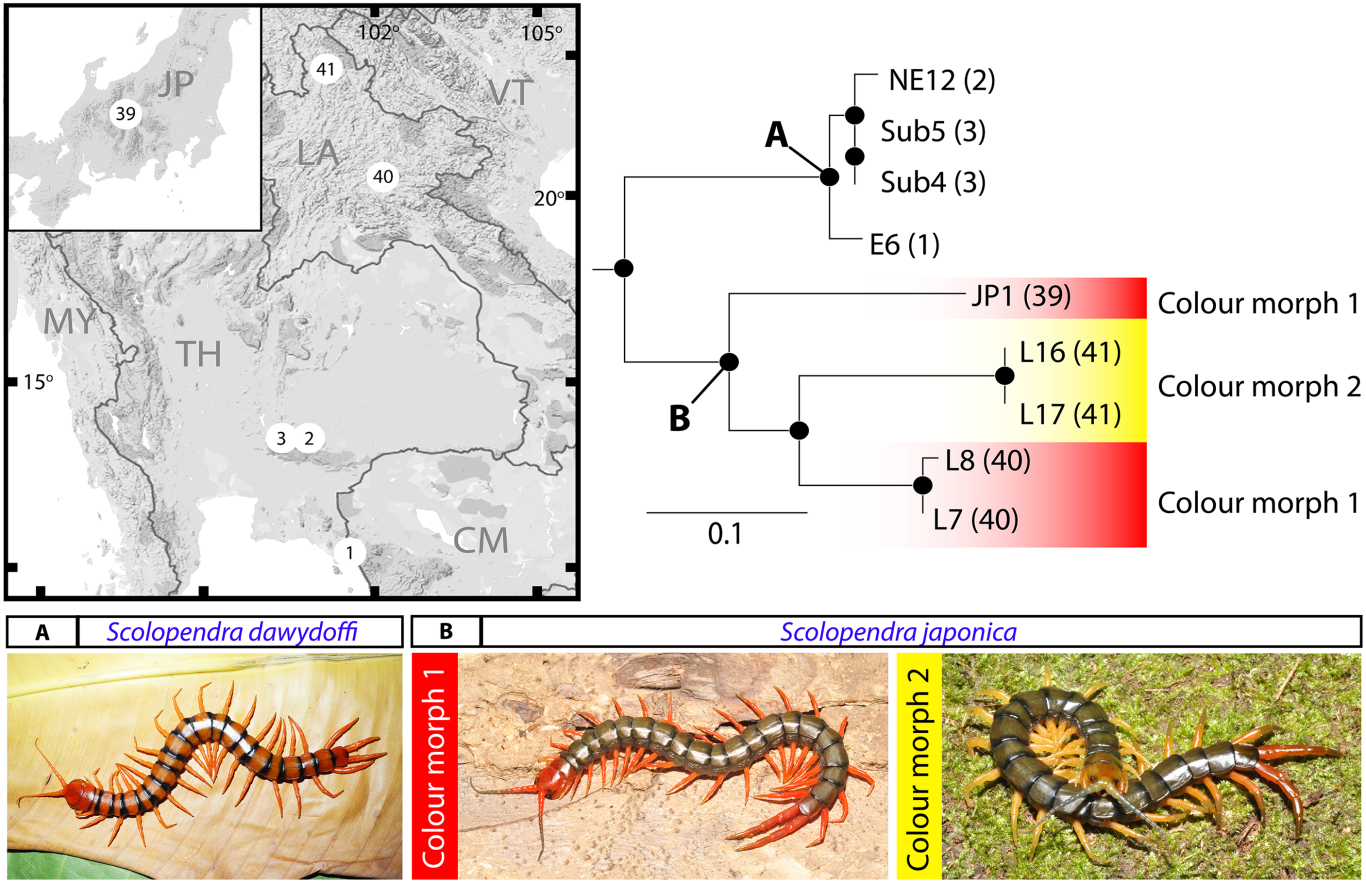
Phylogenetic tree of *S*. *dawydoffi* and *S*. *japonica*. Clade A, *S*. *dawydoffi*, clade B, *S*. *japonica*. In *S*. *japonica*, colour gradients indicate the colour morph of sampled individuals; colour morph 1—greenish body with reddish antenna, cephalic plate and legs; colour morph 2—greenish body with blue antenna, yellowish cephalic plate and legs.

*Scolopendra dawydoffi* and *S*. *japonica* unite as a clade in which both species are reciprocally monophyletic (Fig 5). The taxonomic validity of these two species has been corroborated by strong node support (96/0.99). The corrected distances indicated low genetic diversity among *S*. *dawydoffi* populations as depicted by short branch lengths in the phylogenetic tree (Fig 5; clade A). In case of *S*. *japonica* (Fig 5; clade B), individuals from the sampled populations unite as well-supported clades, the corrected distance within the species being 12.4% and 5.3% in COI and 16S, respectively. A Japanese specimen was resolved as sister taxon to a group composed of the *S*. *japonica* populations from Laos. With regards to colouration, there is no evidence in our collections for *S*. *dawydoffi* exhibiting colour variation. In contrast, *S*. *japonica* has two morphs that can be distinguished by colour of the legs: in colour morph 1, the legs are reddish, whereas colour morph 2 has green-yellowish legs. The Laos fauna includes both colour morphs whereas the specimen from Japan shows similarity to the Laotian population classified as colour morph 1. However, the phylogenetic tree indicated that the colouration does not precisely correlate with genetic affinities in this species.

### Geometric morphometrics

**Fig 6:**
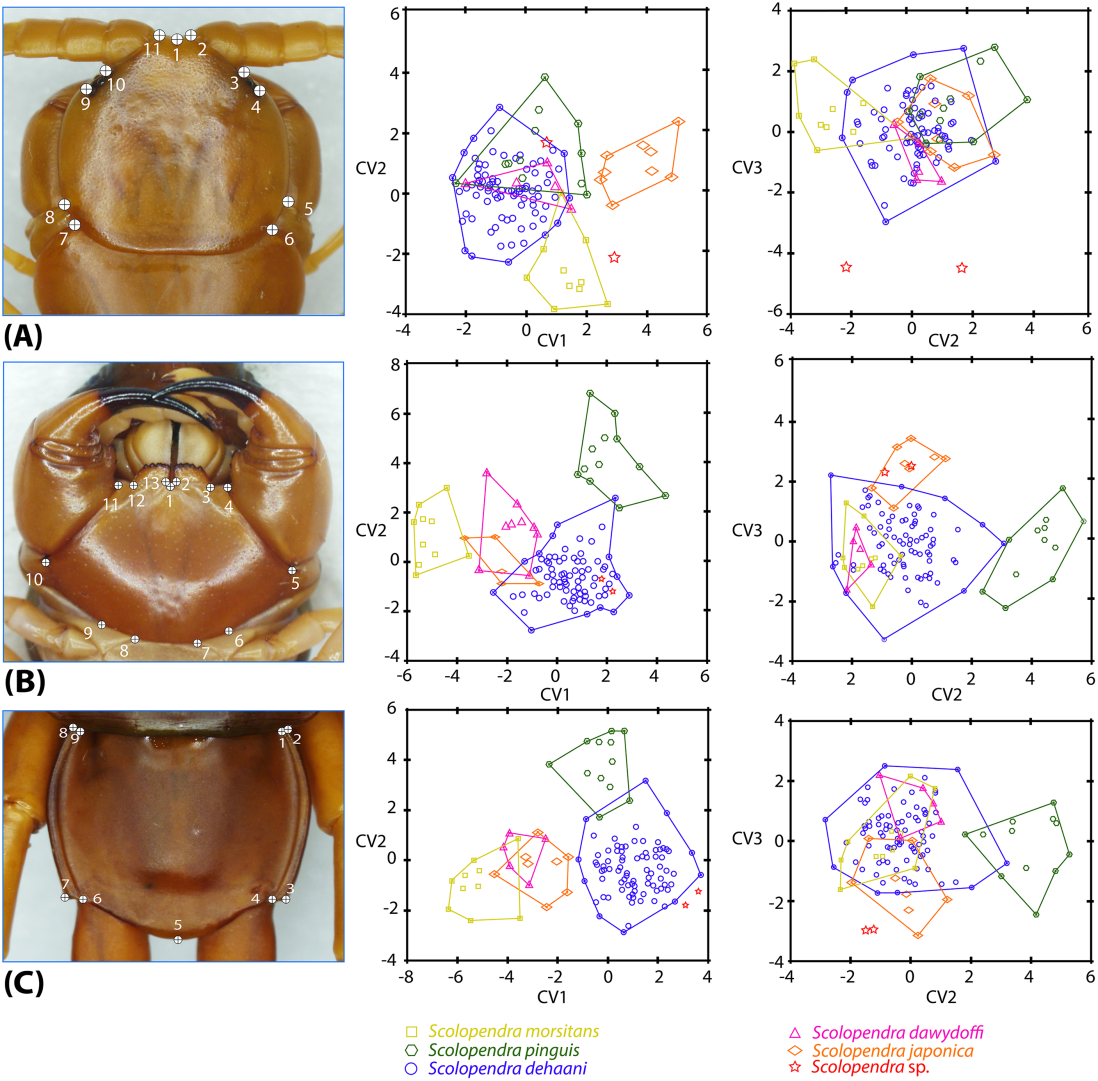
Diagram of landmark locations on three constant characters and the CV plot of individual scores on each CV axis from canonical variates analysis (CVA). A. Cephalic plate; B. Forcipular coxosternite; C. Tergite 21. The CV plots represent the discrimination of classified individuals scored from CV axis comparison, showing comparisons of CV1 and CV2 axes (middle column) and CV2 and CV3 axes (right column)

Six species of *Scolopendra* in the Southeast Asian mainland (excluding *S*. *subspinipes*) have been identified based on morphological taxonomy. Specimen numbers used in this analysis are as follow: five specimens of *S*. *dawydoffi*, 84 specimens of *S*. *dehaani*, eight specimens of *S*. *japonica*, 10 specimens of *S*. *morsitans*, 12 specimens of *S*. *pinguis*, and two specimens of *Scolopendra* sp. The Procrustes ANOVA statistical test found no measurement error in all analyses (*p*<0.0001; S2 Table). CVA plots were performed based on group classified datasets with 10,000 replicates of permutation testing for pairwise distance. Eigenvalue and variable percentages of selected characters are summarized in S3 Table. The three dimensions of CVA plots have been used for species discrimination from shape variation (Fig 6). The discriminant results of *Scolopendra* species from CVA analysis with statistical testing in three selected features are described as follows.

Cephalic plate (Fig 6A): The CV1 axis captured 46.468% of shape variation while the CV2 and 3 axes exhibited 26.269% and 15.575%, respectively. The CVA plot clearly indicated the differentiation of centroid origin in the CV1-CV2 axis. From this CVA plot, all individuals of the same species were grouped together. The clusters of individuals of both *S*. *morsitans* and *S*. *japonica* separated from other species, whereas *S*. *dawydoffi*, *S*. *dehaani* and *Scolopendra* sp. were grouped closely together. In contrast, a poorer discriminant resolution was found in the CV2-CV3 axes, with all species apart from *Scolopendra* sp. overlapping with the range of variation in *S*. *dehaani*. From the statistical result (S4 Table), the *p*-values from permutation tests of Mahalanobis distances among groups supported the discrimination of four *Scolopendra* species, these being *S*. *dehaani*, *S*. *japonica*, *S*. *morsitans* and *S*. *pinguis* (*p*<0.0001). However, no significant difference among groups was indicated by *p*-values of Procrustes distances.

Forcipular coxosternite (Fig 6B): Forcipular coxosternal shape showed appreciable variability in most *Scolopendra* species except in *Scolopendra* sp., although it need be noted that the sample size of the latter species is small. The percentages of variability contributed by the CV1, CV2 and CV3 axes are 54.298%, 30.728% and 7.995%, respectively. The CV1-CV2 pairwise comparison plot showed that individuals of each species show a marked clustering. Variation in the shape range of the forcipular coxosternite in *S*. *japonica* and *S*. *dawydoffi* overlapped and extended into the range of *S*. *dehaani*, whereas *S*. *morsitans* and *S*. *pinguis* are well delineated. In the CV2-CV3 plot, all individuals of *S*. *pinguis* were pooled separately with each other while the remaining species were grouped closely. All individuals of *S*. *morsitans* and *S*. *dawydoffi* were interpolated inside the range of variation of *S*. *dehaani*. From permutation tests (S5 Table) of Mahalanobis distances among groups, *p*-values indicated the distinctness of four *Scolopendra* species, i.e., *S*. *dehaani*, *S*. *japonica*, *S*. *morsitans* and *S*. *pinguis*, with significant support (*p*<0.0001). The Procrustes distances significantly differed only in three examined species, *S*. *dehaani*, *S*. *morsitans* and *S*. *pinguis*.

Tergite 21 (Fig 6C): The shape variation percentages of the three CV axes are as follow: CV1 explained 64.358%, CV2 28.756%, and CV3 5.558%. The CVA analysis exhibited the discriminant centroid origin of CV1-CV2 comparison plot in all examined species. *Scolopendra dehaani*, *S*. *pinguis* and *Scolopendra* sp. depicted wholly or mostly unique ranges of variation. The three remaining species exhibited shape variability against each other. However, the CV2-CV3 comparison plot allowed *S*. *pinguis* and *Scolopendra* sp. to be distinguished from other species. In this comparison, most individuals of *S*. *dawydoffi*, *S*. *japonica* and *S*. *morsitans* were gathered inside the variation of *S*. *dehaani*. The Mahalanobis and Procrustes distances among groups (S6 Table) indicated four distinct species, i.e., *S*. *dawydoffi*, *S*. *dehaani*, *S*. *japonica*, *S*. *morsitans* and *S*. *pinguis*, with statistical support (*p*<0.0001).

**Fig 7:**
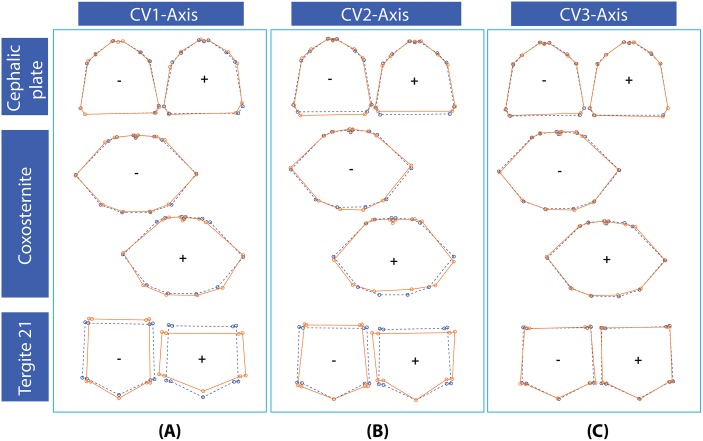
Wireframe diagram from continuous linkage of all landmark positions in three features derived from CVA scores on three axes. CV1, CV2 and CV3 arranged vertically, respectively. In the wireframes, dotted lines represent shape changes relative to CV score moving from both negative and positive directions, solid lines represent the shape consensus in negative and positive groups.

Based on the CVA analyses for each assigned feature, the Eigenvalue and percentage of variance were generated in S3 Table. The variation of the landmarks in the three selected morphological features has been recorded and described as follows:

Cephalic plate: In the CV1 axis, variable reformation was found on Landmarks 1, 2, 3, 4, 5, 6, 7 and 8 (see column A in Fig 7). The position of Landmark 1 shifts posteriorly as CV scores trend positively. This position describes a deeper median sulcus on the anterior part of the cephalic plate. Landmarks 2, 3 and 4 (as well as Landmarks 9, 10 and 11) are displaced medially as CV scores trend from negative to positive. This variation relates to the shape of the cephalic plate, the size of basal antennal article, and relative length of the basal article and the ocelli. The movement of Landmarks 5 and 6 (also 7 and 8) record an increase in width of the posterolateral part of the cephalic plate from positive to negative. In the CV2 axis (column B in Fig 7), variable sites were found to relate to Landmarks 1, 4, 5, 6, 7, 8, 9 and 10. The most extreme change was recognized both in CV negative and positive groups, this being located between Landmarks 5 and 6 (and 7 and 8), affecting length of the cephalic plate. In the CV3 axis (column C in Fig 7), only six landmarks showed slight variability, these being Landmarks 1, 2, 3, 6, 7 and 8 in both CV positive and negative score datasets. This variation again impacted on the length of the cephalic plate.

Forcipular coxosternite: In the CV1 axis, shape variation was detected in all landmark points (column A). Movement in Landmarks 3–12 describes the shape of the coxosternite in both CV negative and positive groups. The forcipular coxosternite in the CV positive group is relatively shorter than in the negative. Landmarks 3 and 4 (also 11 and 12) shift laterally from negative to positive, describing a broadening of the anterolateral corner of the coxosternite. Landmarks 6, 7, 8 and 9 moves anteriorly from negative to positive. In the case of the CV2 axis (column B in Fig 7), the outline between Landmarks 12–13 and 2–3 showed variation. Specifically, landmarks describing the course of the anterior parts of the forcipular coxosternite shift from more curved to straighter towards the positive group. Moreover, the posterior part of the forcipular coxosternite also showed high variability at Landmarks 7 and 8, which shift anteriorly from negative to positive groups. Simultaneously, the coxosternal condyles are situated more posteriorly from negative to positive groups. From this variability, the outline of the forcipular coxosternite in the CV positive group is more trapezoidal and straighter / more transverse across both its anterior and posterior parts whereas the CV negative group exhibited a more curved diamond shape on its anterior and posterior parts. From the CV3 axis (column C in Fig 7), the shape variation of the forcipular coxosternite in both the CV negative and positive groups showed only slight change, this being captured by Landmarks 2, 3, 4, 5, 6, 7, 8 and 10.

Tergite 21: In the CV1 axis, shape variation exhibited substantial changes in both CV negative and positive groups (Column A in Fig 7). Tergite 21 depicts relatively elongate versus broad rectangular shapes in the CV negative and positive groups, respectively. According to Landmark 5 in particular, the posterior margin of tergite 21 shifts posteriorly from positive to negative, and combined with movements of other landmarks corresponds to the change in length: width ratio of the tergite. Landmarks 1, 2, 8 and 9 shift laterally from negative to positive groups, whereas Landmarks 2, 3, 7 and 8 shift so that the tergite transforms from longer than wide to the reverse. In CV 2 axis (column B in Fig 7), a stable landmark was identified in Landmark 5, which showed consistency in both CV extremes. However, the visualized shape outline of tergite 21 also reflected the distinctness between CV populations, similar to the case for the CV1 axis. The CV positive group shows a displacement of Landmarks 3 and 4 (also 6 and 7) so that the posterior margin of the tergite changes from a broad V-shape to having the short extent delimiting the margination being transverse, and then flexing to its V-shaped extent. In the CV3 axis (column C in Fig 7), the shape of tergite 21 showed less variation than did the CV1 and CV2 axes. Landmarks 1, 3, 5, 7, 8 and 9 all exhibited variability on this axis. In the CV negative group, tergite 21 has the lateral margin (between Landmarks 2 and 3 / 7 and 8) more strongly diverging posteriorly than in the CV positive group.

From the results of discriminant function analysis among classification categories (Table 6), five assigned categories of morphometric samples were confirmed by the percentage of correct classification in two features, the forcipular coxosternite and tergite 21 (>80% in all categories). However, the percentage of correct classification based on variation in the cephalic plate was lower (<50%) in *S*. *dawydoffi* and *Scolopendra* sp. The cross-validation of discriminant function analysis showed a low percentage of correct classification in all sampled characters and taxa except for *S*. *dehaani*, which received 89% correct classification when defined by the forcipular coxosternite and tergite 21.

**Table 6:** Results of CV discriminant function in three selected characters; the total number and percentage of correction of leave-one-out cross validation tests in CV discriminant function are in parentheses.

Character	Species	*Scolopendra dawydoffi*	*Scolopendra dehaani*	*Scolopendra japonica*	*Scolopendra morsitans*	*Scolopendra pinguis*	*Scolopendra* sp.	Total number	% of correction
Cephalic plate									
	***Scolopendra dawydoffi***	4 (2)	1 (3)	0 (1)	0 (2)	0 (2)	3 (3)	4 (2)	50 (18)
	***Scolopendra dehaani***	7 (11)	75 (71)	0 (1)	2 (7)	7 (13)	0 (3)	75 (71)	83 (67)
	***Scolopendra japonica***	0 (4)	0 (3)	8 (4)	0 (4)	0 (6)	1 (3)	8 (4)	89 (17)
	***Scolopendra morsitans***	0 (5)	1 (2)	0 (5)	10 (5)	0 (5)	0 (4)	10 (10)	91 (19)
	***Scolopendra pinguis***	0 (7)	2 (8)	0 (6)	0 (6)	12 (5)	0 (1)	12 (5)	86 (15)
	***Scolopendra* sp.**	1 (2)	0 (2)	0 (1)	0 (1)	0 (3)	1 (0)	1 (0)	50 (0)
Forcipular coxosternite									
	***Scolopendra dawydoffi***	5 (2)	0 (3)	0 (1)	0 (1)	0 (0)	1 (0)	5 (2)	84 (29)
	***Scolopendra dehaani***	3 (8)	79 (74)	2 (6)	0 (0)	1 (4)	0 (0)	79 (74)	93 (80)
	***S*. *japonica***	0 (0)	0 (2)	8 (8)	0 (1)	0 (1)	0 (0)	8 (8)	100 (67)
	***Scolopendra morsitans***	0 (3)	0 (1)	0 (2)	10 (7)	0 (4)	0 (1)	10 (7)	100 (39)
	***Scolopendra pinguis***	0 (3)	0 (3)	0 (2)	0 (5)	12 (12)	0 (1)	12 (12)	100 (46)
	***Scolopendra* sp.**	0 (2)	0 (2)	0 (1)	0 (1)	0 (2)	2 (0)	2 (0)	100 (0)
Tergite 21									
	***Scolopendra dawydoffi***	5 (5)	0 (0)	0 (4)	0 (3)	0 (0)	0 (0)	5 (5)	100 (42)
	***Scolopendra dehaani***	1 (1)	79 (81)	1 (3)	0 (0)	1 (5)	1 (1)	81 (81)	95 (89)
	***Scolopendra japonica***	0 (2)	0 (1)	8 (6)	0 (2)	0 (1)	0 (0)	8 (6)	100 (50)
	***Scolopendra morsitans***	0 (7)	0 (0)	0 (4)	10 (3)	0 (0)	0 (0)	10 (3)	100 (21)
	***Scolopendra pinguis***	0 (1)	0 (3)	0 (1)	0 (0)	12 (11)	0 (0)	12 (11)	100 (69)
	***Scolopendra* sp.**	0 (0)	0 (2)	0 (1)	0 (1)	0 (2)	2 (2)	2 (2)	100 (25)

## Discussion

### The diversity of *Scolopendra* in mainland Southeast Asia

The field survey in this study identified the occurrence of *S*. *japonica* in mainland SE Asia, its character data conforming to a recent taxonomic review [30]. The distribution range of all species has been refined and it is now possible to make inferences on several species usually reported as regional widespread species [22]. In this study, seven *Scolopendra* species have been found in both natural and anthropogenic areas. Previous records of *Scolopendra* in SE Asia [42, 90, 91] indicated that there are three additional species that can potentially be found in this area: *S*. *gracillima* Attems, 1898 [92], *S*. *calcarata* Porat, 1876 [93], and *S*. *hardwickei* Newport, 1844 [94]. However, very few specimens were reported in the relevant studies and the species listed above were treated as likely introductions [42]. In mainland SE Asia, *S*. *dehaani* has been found to be the dominant, widespread species throughout the sampling territory, whereas *S*. *subspinipes* (*S*. *subspinipes subspinipes* of most previous studies) is rarely found, as conforms to previous work [91, 95, 96]. In contrast to those species, some species seem to be endemic and have scattered distributions and sparse populations in nature, i.e., *S*. *dawydoffi*, *S*. *japonica*, *Scolopendra* sp. and *S*. *pinguis*. From phylogenetic analysis, the regional populations of some examined species such as *S*. *dehaani*, *S*. *morsitans* and *S*. *pinguis* suggested the genetic affinities among geographically neighboring members, infraspecific structure that might be affected by the geographical richness of the region. Several areas have been promoted as corridors for dispersal or land bridges [1, 97, 98], and the mechanisms that sculpt the gene pool among regional populations may derive from these boundaries, as has been proposed for other invertebrates and vertebrates [99–101].

#### Morphological discrimination among nominal species

In the past, the status of several scolopendrid genera such as *Cormocephalus* Newport, 1844 [102], *Trachycormocephalus* Kraepelin, 1903, and *Arthrorhabdus* Pocock, 1891 [103] has been debated relative to their distinction from *Scolopendra*. Because of its morphological variability and distribution, several species have now been revised [30] while some of them were relegated to synonymy with particular members of *Scolopendra* [104]. From the available collections, sourced from different geographical localities throughout mainland SE Asia, some examined species such as *S*. *japonica* and *S*. *dawydoffi* exhibited morphological variation among their populations. Diagnostic characters such as the number of teeth on the coxosternal tooth-plates, tergite margination, and the number of spines on the ultimate leg prefemur showed high variability, as has previously been recognized in various species of *Scolopendra* [30]. Overlapping proportions and a shortage of diagnostic characters are more pervasive challenges in Scolopendromorpha, affecting other genera as well, such as *Otostigmus* Porat, 1876 [93], *Digitipes* Attems, 1930 [105], *Rhysida* Wood, 1862 [106] and *Cryptops* Leach, 1816 [23]. In our examined species, *S*. *dehaani* exhibited morphological consistency of its diagnostic characters in all populations. The morphology of *S*. *dawydoffi* and *S*. *japonica* is most similar, as demonstrated by the flattened ultimate leg in both species, but they can be distinguished by the extent of the sternal paramedian sutures. A taxonomic issue for *S*. *dawydoffi* is its morphological similarity with another tropical-temperate species, *S*. *multidens* Newport, 1844. Only one character, the male genital segment without appendages (gonopods), has been proposed to be diagnostic for *S*. *multidens* [37], but we have found that this character also occurs in *S*. *dawydoffi* from Thailand, and it is furthermore shared by another insular species, *S*. *hainanum* Kronmüller, 2012, from Hainan Island, China. We presently decline to synonymise *S*. *dawydoffi* with *S*. *multidens* because preliminary phylogenetic results suggest they are genetically distinct [107]. Species nomenclature uses geographical information, *S*. *dawydoffi* being distributed in the Indochina subregion whereas *S*. *multidens* is distributed in Taiwan and other part of the East Asian temperate zone. The problem of uncertain diagnostic characters was also recognized between *S*. *pinguis* and *S*. *gracillima*, as discussed in recent taxonomic reviews [22]. Solving such taxonomic problems in *Scolopendra* will likely require comprehensive description and DNA sequence data for samples from across the distributional ranges of the relevant nominal species.

#### Molecular species delimitation

The phylogenetic tree from partial gene analysis showed informative resolution for the purpose of species delimitation. The clade corresponding to the subfamily Scolopendrinae showed reasonable support in BI analysis for its monophyly, but *Scolopendra* itself cannot be defended as monophyletic. Even for the present sampling of SE Asian species, the exemplar species of *Cormocephalus* nested within *Scolopendra* rather than resolving as sister group to a monophyletic *Scolopendra*. This is not an especially surprising finding because recent broad-scale molecular and morphology-based phylogenetic analyses of Scolopendromorpha found *Scolopendra* to be paraphyletic or polyphyletic with respect to other genera of Scolopendrini [51]. The genus in its traditional (and current) guise is diagnosed by a combination of characters (e.g., the cephalic plate overlapping T1, tooth-plates shorter than those of *Arthrorhabdus*, presence of tarsal spurs, accessory spurs on the pretarsus), all of which are likely symplesiomorphies for Scolopendrini, without an obvious autapomorphy that would signal monophyly.

In contrast to the unsettled status of the genus, part of a more pervasive problem with the diagnoses and delimitation of genera in Scolopendridae [49], our analyses provide clearer insights into the phylogenetic status of species. In this study, the former subspecies of *S*. *subspinipes* sensu Lewis 2010 (here determined as *S*. *dehaani*, *S*. *subspinipes*, *S*. *japonica* and *S*. *dawydoffi*) have been taxonomically validated, as was suggested by the most recent morphological classification [30]. However, comprehensive description of their morphology is needed to improve species discrimination.

Previously, evaluation of barcode gaps in members of *Scolopendra* has been undertaken only in three species: *S*. *cingulata*, *S*. *cretica* Lucas, 1853 [108] and *S*. *canidens* Newport, 1844 [94]. These showed an average interspecific variation between 13.4–14.8% in the universal COI barcode region [26]. Species of *Scolopendra* in the present analysis exhibited greater genetic distance than in previous studies, the relative morphospecies comparison depicting genetic distances from 15% to 19.9% in COI. The high genetic distance both between and within species might be useful for species delimitation of *Scolopendra*, being similar to previous evidence from phylogenetic studies of scolopendromomorphs, including *Digitipes* (Indian populations; 14.2–19.4% for COI [47], Burmese-Indian populations; 14.5–21.3% [52]) and *Cryptops* (Pacific island populations; 19.8–23.7% [48]). In the case of intraspecific variation, genetic distances in this analysis were 8.6% for COI in two cosmopolitan species (*S*. *dehaani* and *S*. *morsitans*) whereas the more narrow-ranged species, *S*. *pinguis*, *S*. *japonica* and *Scolopendra* sp., show greater genetic diversity (18.3%, 12.2% and 12.4% in COI, respectively).

Genetic distance in *S*. *pinguis* in particular is indicated by branch lengths in the phylogenetic tree and high intraspecific variation in COI, 16S and 28S genes (Table 5). This pattern is often ascribed in invertebrates to the process of cryptic speciation [3, 11, 47, 109, 110]. For this reason, the sample of *S*. *pinguis* might contain more than one species or may at least be in the early stage of cryptic speciation. *Scolopendra morsitans*, a cosmopolitan pantropical species, showed two lineages from their genetic structure. An eastern population that shows low genetic diversity among its populations seems to indicate high genetic transfer in this species in this area because this situation also occurred in another widespread species, *S*. *dehaani*, in which all populations exhibited low genetic diversity. Dispersal mechanisms among these widespread species are of interest and may be clarified by population genetic and demographical historical studies as have been undertaken for some other *Scolopendra* species [26, 27]

### Taxonomic validity of some *Scolopendra* members in SE Asia

Currently, the species diversity of *Scolopendra* in Southeast Asia comprises 13 species that are distributed in the mainland and insular faunas [21]. Among them, morphological examination is adequate for species delimitation in some species, such as *S*. *morsitans* and *S*. *dehaani*. However, there are other species that show high morphological variability, indeed more than previously estimated, such as *S*. *dawydoffi*, *S*. *japonica* and *S*. *pinguis*. As noted above, their variability might overlap with other related species in this region such as *S*. *multidens* (in the case of *S*. *dawydoffi*) and *S*. *gracillima* (in the case of *S*. *pinguis*) [29]. *Scolopendra dehaani* was originally established as a full species [56] but because of its morphological similarity with *S*. *subspinipes*, subsequent taxonomists treated it as a subspecies of *S*. *subspinipes*, until it was only recently revalidated as a separate species [30]. Another two former subspecies of *S*. *subspinipes*, *S*. *dawydoffi* and *S*. *japonica* (previously known as *S*. *subspinipes cingulatoides* Attems, 1938, and *S*. *subspinipes japonica* Koch, 1878, respectively), have likewise most recently been elevated to the ranks of separate species [30]. Our molecular examination confirmed the validation of these two species, not the least because they are resolved as more closely related to each other than either is to *S*. *subspinipes* (Figs 1 and 3), and was supported by new additional characters for species discrimination. For these reasons, we consider that three former subspecies of *S*. *subspinipes* sensu Lewis, 2010 are valid species, as likewise determined based on external morphology alone [30].

### Phylogeography of *Scolopendra* in mainland SE Asia

Phylogeography has been introduced for centipede systematic studies in the past decade [19, 20, 27, 109]. Geological events in the past that potentially bear on the distribution range of these animals include the drift and collision of former fragments of Gondwana during the Jurassic [46], micro-refugia in the last glacial period during the Pleistocene [26, 27], and the debated hypothesis of biotic shuttle for some insular centipedes [48]. Phylogenetic results in this study depict genetic relationships of some *Scolopendra* populations that are congruent with geographical barriers in mainland Southeast Asia. These findings may relate to the two sub-regional faunas of the Indo-Burmese biodiversity hotspot, Indochina and Malesia [1, 111–113]. The genetic structure of *S*. *dehaani* populations suggested a separation into three lineages. The CPB population occupies the northern, central and some western elements of Thailand while the MRB population contained the entire eastern element of the Indochina sub-region, including the northeast and east of Thailand, Laos and Cambodia. The separation between these two major populations is located on the western margin of Korat Plateau along the Phetchabun, Dong Paya Yen and Sankambeng mountain ranges. Uplift of the Korat Plateau has been estimated to date to the middle-late Triassic [114, 115]. This plateau is delimited by the mountain block between northeastern Thailand and Cambodia, which is likely to have initiated speciation and divergences between these two faunas, as exemplified by amphibians [16] and reptiles [116]. However, our analysis groups together the entire population of *S*. *dehaani* from northern Laos to the eastern coast of Thailand, demonstrating less sensitivity to vicariance than in some other animal groups.

In the case of the CPB population, the northwest and central Thai samples were united as a core group. The one sample from the upper northern region exhibited genetic difference from the rest, being resolved as the basal clade of the CPB population. The genetic distance may reflect the genetic transfer among neighbouring populations along a geographical gradient. The parallel mountain range along the western and northern parts of Thailand may not limit the dispersal of this population only to the northwestern part of Thailand. Collision of the Indian subcontinent with the Laurasian plate during the Eocene (55 Mya) [98, 117, 118] is likely to have contributed to dispersal of *S*. *dehaani*, which is known from India [119]. Other elements of the SE Asian and Indian biotas, both extinct [120] and extant [47], show similar patterns of diversification. Moreover, a hypothesis that refers to some Indian centipede taxa dispersing into SE Asia is consistent with molecular dating and genetic composition among populations [52].

Similar to the previous population, the LTR population is divided into two main groups. The first covered the population along the Isthmus of Kra, which is assumed to be a transitional zone for animals in this region [111, 121, 122]. The second lineage is the lower Tenasserim Range population, which extends from the central part of southern Thailand to the Malay Peninsula. These two minor populations were separated by the mountain valley between the Phuket Range in the west coast and the Titiwangsa Range in the lower east coast of the Thai-Malay Peninsula. Sea level fluctuation in the South China Sea and its role in habitat change/loss has been discussed as a factor impacting on population-level patterns in this region [112, 123–127], and also contributed to exposure of the Sunda Shelf along the coastal areas of Indochina and the Malay Archipelago [101, 112, 128–131]. These geological events seem to be driving mechanisms for the migration of both flora [132] and fauna [16, 111, 116, 133, 134] in this area. Our result showed that one sample from the eastern coast of Thailand exhibited genetic affinity with the entire southern population. This result is congruent with data from taxonomic studies on other animal groups that report relationships between populations from the same two regions, including butterflies [135] as well as centipedes [42, 136]. These repeated patterns are suggestive of a cause that is linked to the geographical history of Sundaland. However, passive dispersal from anthropogenic activity is also a possibility because of the habitat preference of this species, *S*. *dehaani* often being collected in disturbed sites with human modification.

Contrary to the widespread *S*. *dehaani*, the rare species *S*. *pinguis* exhibited high genetic diversity among its populations. The updated distribution from this study showed a limit to dispersal along the northwestern and northeastern mountain ranges of mainland SE-Asia. Genetic distances indicated that population structure relates to geography in that western, central and eastern populations are differentiated along the northern region. Genetic variability likewise reported in other morphologically conservative groups of vertebrates and invertebrates has often been found to signal cryptic species, as exemplified by amphibians [137], molluscs [3], diplopods [12], and even in some scolopendrid centipedes [47]. Accordingly, some of the distinct populations of *S*. *pinguis* in the west should be monitored to test the hypothesis that cryptic species may be involved.

In case of *S*. *japonica*, the phylogenetic result showed two regional populations. This genetic relationship provides evidence for shared animal diversity between the eastern coast of the Palearctic (here sampled by Japan) and Indo-Malay ecozones, as reported in other invertebrates, e.g. dragon flies [138], scorpions [139], and spiders [140, 141]. Populations within *S*. *dawydoffi* depict close relationships to each other (i.e., short branch lengths). However, the population from the east coast of Thailand was resolved as sister group to the remaining conspecific populations. The continuous geography of this area may facilitate genetic transfer among these populations, as is likewise the case for populations of *S*. *morsitans* from Northeastern and Eastern Thailand and Cambodia. The ecological richness of this area likely contributes to it being a migration route for some organisms throughout the Indochina sub-region [142]. However, the precise distribution range of *S*. *dawydoffi* is unknown (records are scattered in Laos and Vietnam) and additional material will be needed to reveal the fine detail of its genetic structure.

### Colouration patterns of *Scolopendra*

The colour variation on animal bodies has been promoted as an interesting topic for evolutionary studies of various kinds of organisms, such as nudibranchs [143], land snails [144], and butterflies [145], as well as in centipedes [26]. Recently, molecular phylogeny has been used to explain the relationship between two different colour morphs distributed sympatrically with each other [26]. In our survey, colour variation in populations was recorded for all examined species, *Scolopendra* being the only centipede genus in the biota that shows highly diverse patterns of colour morphs [34, 35]. Several species exhibited extreme difference as bright or contrasted colour morphs between populations. The number of patterns differs in various species [95], for example *S*. *laeta* Haase, 1887 from Australia exhibited five colour morphs [34], two colour morphs of *S*. *cingulata* were found in the Mediterranean [26], and four colour morphs have been documented in *S*. *morsitans amazonica* Bücherl, 1946 [146] (= *S*. *morsitans*) from Africa [147]. Moreover, the significance of colour variation in several species has been debated in the literature with regards to its taxonomic value. In previous studies, authors usually assigned the different colour morphs of centipedes to new species [148, 149] or treated them as subspecies or varieties of species [59]. Recently, several taxonomic revisions argued against this approach. The number of fresh specimens for examination in several species is limited, and when colours have faded in preserved museum specimens the taxonomic utility of colour is compromised. For these reasons, several nominal species in the past, which had been identified by colouration pattern, have fallen into synonymy with other widespread species [30, 35]. However, our study revealed the relationship of these morphological changes to the genetic structure of some species, in several cases showing that colour morphs correspond to monophyletic groups.

In this study, the species with most diverse colour morphs is *S*. *dehaani*. This centipede exhibited five colouration patterns. Colour morphs 2, 3 and 4 are standard colour morphs which are usually found in all populations of *S*. *dehaani* whereas colour morphs 1 and 5 are specific to only two regional populations, CPB and TLH. Environmental factors such as humidity and other habitat characteristics were assumed to affect this variability. Comparable variability in colouration has been reported and discussed in systematic studies on tropical organisms, e.g., snakes [150], amphibians [151], and sea cucumbers [152]. These results suggest a correlation between colouration and such factors such as habitat type [153] and predation [154].

The distinct colouration patterns in *S*. *pinguis* populations differ from other species in our analysis. In general, juvenile *Scolopendra* show different colour patterns compared to adult stages, e.g., in *S*. *morsitans* and *S*. *dehaani*. In the case of *S*. *pinguis*, however, colouration patterns are consistent through an observed series of post-embryonic developmental stages, such that paedomorphosis may account for this variation. Ontogenetic data such as these have been suggested as necessary to clarify some taxonomic problems in scolopendromorphs [155, 156].

The remaining examined species, *S*. *morsitans* and *S*. *japonica*, exhibited specific colour morphs in different populations. Interestingly, the black and reddish patterns found in *S*. *morsitans* were also reported in African and Australian populations [34, 157]. The pattern of colouration change is specific to particular morphological characters such as the cephalic plate and tergite of the ultimate leg-bearing segment [34, 95], as often noted in taxonomic descriptions of *Scolopendra*. According to the colour variation of these samples, we can separate the colouration pattern among SE Asia *Scolopendra* into either monochrome or dichrome (Table 1), following similar descriptions in some previous *Scolopendra* studies [37]. The colour polymorphism of *Scolopendra* populations might be useful as a model for molecular ecology and evolutionary studies in SE Asian centipedes.

### Taxonomic implications of shape variation

Shape variation tests in morphology have been widely used in fossilized material because of a frequent shortage of morphological characters for species identification [158–160]. These techniques have also been applied to systematic study of varied organisms for species discrimination [3, 161, 162]. In this study, the three surveyed features show results that appear to be useful as taxonomic signal for species-level taxonomy, as exemplified by plots from canonical variates analysis (Fig 6) and CV discriminant functions (Table 6). Each of the examined characters had been documented in some scolopendrid literature over the past century [29, 61], but this study showed the first indications of taxonomic value of these features based on their discrete shape variation among *Scolopendra* species. Shape variation in tergite 21 seems to be the most variable of the features explored in our analysis and, indeed, this character has been used as diagnostic for some scolopendrid species, e.g., *Cormocephalus cupipes* Pocock, 1891 [102] and *Otostigmus caudatus* Brölemann, 1902 [163]. To reduce confounding effects and improve accuracy, further shape analysis techniques such as combined outline and semi-landmark methods that have been used in scutigeromorph centipedes [31, 32], as well as meristic measurements [33], are likely to be promising topics for further study.

## Conclusions

Taxonomy of scolopendrid centipedes at the species level has classically used external morphology, and *Scolopendra* is no exception. The phenotypic characters used herein for species delimitation in SE Asia (Table 1) are ones that have been employed by experts in scolopendrid taxonomy over many decades. Despite their widespread usage, the taxonomic reliability of these characters has at some time been called into question because they depict variability within particular scolopendrid species [30, 35, 78, 164]. External morphology offers an accessible toolkit for identifying scolopendrid species but whether or not these entities (morphospecies) correspond to monophyletic groups has usually not been clear because taxonomy has usually been conducted separately from phylogenetic analysis. At the same time, *Scolopendra* has been subjected to perennial discussion about whether various taxa are species, subspecies or infraspecific variants undeserving of formal taxonomic recognition, a situation best exemplified by the unstable taxonomy of *S*. *subspinipes* and the dozens of nominal species now considered as junior synonyms of *S*. *morsitans*.

We have aimed to test the status of morphospecies in *Scolopendra* in SE Asia by applying additional data sources, specifically DNA sequences and geometric morphometric approaches to shape analysis. The results of these tests demonstrate that morphospecies are monophyletic groups supported by strong node support in probabilistic molecular analyses. Furthermore, these same formulations of species can be identified by statistical morphometric methods using such features as the shape of the forcipular coxosternite and the tergite of the ultimate leg-bearing segment. These shapes had generally not been used in prior taxonomy, possibly because the subtle differences between species are less amenable to qualitative description than to quantitative analysis. We are hesitant to generalize on whether the concordance between species delimited by external characters and molecular tree topologies seen in this set of *Scolopendra* species is representative of centipede taxonomy more broadly. At least some previous analyses, such as for the Neotropical scolopendromorph genus *Newportia* Gervais, 1847 [165], have found that at least some morphospecies correspond to para- or polyphyletic groups based on molecular phylogeny [156]. Other analyses, as for Indian species of the scolopendrid *Digitipes*, have found a better match between morphological and molecular estimates of species, though some instances of cryptic species are likely [47, 166]. Finally, our study suggests that colour variation and genetic diversity of SE Asian *Scolopendra* populations can likely be attributed both to natural and anthropogenic pressures that affected genetic composition and ontogeny of these centipedes.

## Supporting Information

S1 Fig Phylogenetic tree based on concatenation of three partial gene analysis. Support values of bootstrap and posterior probability were given at each node.
(TIF)

S1 Table Sample size, CUMZ registration numbers and collecting localities of specimens in geometric morphometric analysis under Landmark base method. (DOCX)

S2 Table Error measurement in three selected features of *Scolopendra*, shapes of the cephalic plate, forcipular coxosternite and tergite 21, in Procrustes ANOVA analyses. (DOCX)

S3 Table Eigenvalue and percentage of variance from Canonical variance analysis of the cephalic plate, coxosternite and tergite 21. (DOCX)

S4 Table Mahalanobis and Procrustes distances of cephalic plate shape from pairwise-comparison of species classifiers in Canonical variance analysis. (DOCX)

S5 Table Mahalanobis and Procrustes distances of forcipular coxosternite shape from pairwise-comparison between species classifiers in Canonical variance analysis. (DOCX)

S6 Table Mahalanobis and Procrustes distances of tergite 21 shape from pairwise-comparison between species classifiers in Canonical variance analysis. (DOCX)
