# Correction: The Centipede Genus *Scolopendra* in Mainland Southeast Asia: Molecular Phylogenetics, Geometric Morphometrics and External Morphology as Tools for Species Delimitation

**DOI:** 10.1371/journal.pone.0139182

**Published:** 2015-09-22

**Authors:** The PLOS ONE Staff

## Notice of Republication

This article was republished on September 8, 2015, to correct the order of Figs. 3–5. Please download this article again to view the correct version. The originally published, uncorrected article and the republished, corrected article are provided here for reference.

## Supporting Information

S1 File Originally published, uncorrected article. (PDF)

S2 File Republished, corrected article. (PDF)
