# Supplementary figures and images for: The Centipede Genus Scolopendra in Mainland Southeast Asia: Molecular Phylogenetics, Geometric Morphometrics and External Morphology as Tools for Species Delimitation
Source: PLoS One. 2015 Aug 13;10(8):e0135355. doi: 10.1371/journal.pone.0135355 (PMC4536039; doi:10.1371/journal.pone.0135355)

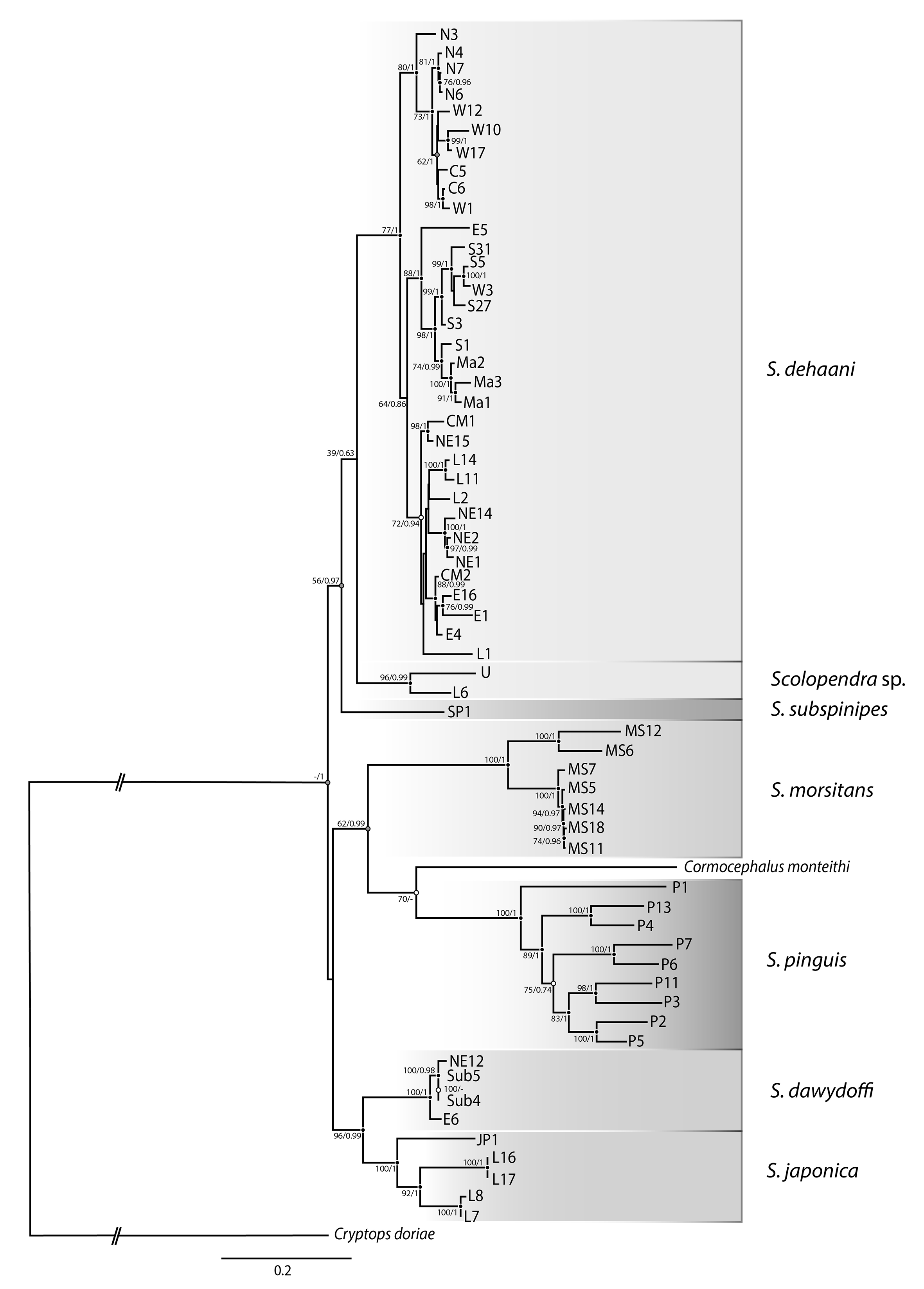

Supplement: S1 Fig — Support values of bootstrap and posterior probability were given at each node. (TIF) [file pone.0135355.s001.tif]
