# Supplementary material for: The Centipede Genus Scolopendra in Mainland Southeast Asia: Molecular Phylogenetics, Geometric Morphometrics and External Morphology as Tools for Species Delimitation
Source: PLoS One. 2015 Aug 13;10(8):e0135355. doi: 10.1371/journal.pone.0135355 (PMC4536039; doi:10.1371/journal.pone.0135355)
Supplement: S1 Table — (DOCX) [file pone.0135355.s002.docx]

**S1 Table**

| **Sample code** | **Locality** | **Species** | **CUMZ Nos.** | **GPS coordinate** | **Number of specimen** |
| --- | --- | --- | --- | --- | --- |
| M001 | Wat Huailat, Phu Ruea, Loei | *S. dehaani* | 00241 | 17°27'00.4"N 101°24'45.4"E | 1 |
| M002 | Mueang Khong, Champasak, Laos | *S. dehaani* | 00242 | 14°07'03.7"N 105°51'15.0"E | 1 |
| M003 | Hub Pa-Tat, Larnsak, Uthaithani | *S. dehaani* | 00243 | 15°22'37.4"N 99°37'51.9"E | 1 |
| M004 | Wat Tham Phuthakodom, Sinakharin, Phatthalung | *S. dehaani* | 00244 | 7°33'37.2"N 99°53'07.3"E | 2 |
| M005 | Ban Ka Soam, Attapue, Laos | *S. dehaani* | 00245 | 14°49'15.8"N 106°49'05.0"E | 1 |
| M006 | Ban Xai Na Pho, Champasak, Laos | *S. dehaani* | 00246 | 15°25'28.3"N 106°36'34.8"E | 1 |
| M007 | Ban Dong Savanh, Phang Khon, Sakon Nakhon | *S. dehaani* | 00247 | 17°28'17.9"N 103°30'12.6"E | 1 |
| M008 | Kaeng Lamduan, Ubon Ratchathani | *S. dehaani* | 00248 | 14°26'15.0"N 105°06'06.7"E | 2 |
| M009 | Ban Dan Chang, Tha Kantho, Kalasin | *S. dehaani* | 00249 | 14°49'29.3"N 99°41'36.5"E | 2 |
| M010 | Ron Waterfall, Khlong Thom, Krabi | *S. dehaani* | 00250 | 7°56'07.4"N 99°12'37.5"E | 3 |
| M011 | Sai Rung Waterfall, Takua Pa, Krabi | *S. dehaani* | 00251 | 7°26'26.2"N 99°48'47.5"E | 3 |
| M012 | Si Chang Island, Chonburi | *S. dehaani* | 00252 | 13°09'08.1"N 100°48'29.3"E | 1 |
| M013 | Tham Khao Bin, Ratchaburi | *S. dehaani* | 00253 | 13°35'35.6"N 99°40'02.3"E | 1 |
| M014 | Wat Khao Chakan, Srakaeo | *S. dehaani* | 00254 | 13°39'38.0"N 102°05'02.7"E | 1 |
| M015 | Tham Rue Si, Kantang, Trang | *S. dehaani* | 00255 | 7°28'35.9"N 99°29'04.0"E | 1 |
| M016 | Bang Ban, Ayutthaya | *S. dehaani* | 00256 | 14°21'51.4"N 100°29'22.3"E | 1 |
| M017 | Wat Huai Lung, Banphot Phisai, Nakhon Sawan | *S. dehaani* | 00257 | 15°55'29.6"N 99°52'28.0"E | 1 |
| M018 | Wat Khao Maidaeng, Sriracha, Chonburi | *S. dehaani* | 00258 | 12°56'56.0"N 101°02'11.9"E | 2 |
| M019 | Chao Mai Beach, Sikao, Trang | *S. dehaani* | 00259 | 7°26'27.8"N 99°20'45.0"E | 1 |
| M020 | Wat Tham Suea, Krabi | *S. dehaani* | 00260 | 8°07'26.1"N 98°55'27.2"E | 1 |
| M021 | Surin Islands, Phangnga | *S. dehaani* | 00261 | 9°26'58.4"N 97°52'37.8"E | 1 |
| M022 | JPR Stone park, Kraburi, Ranong | *S. dehaani* | 00262 | 10°29'36.7"N 98°54'35.7"E | 1 |
| M023 | Lanta Island, Krabi | *S. dehaani* | 00263 | 7°29'55.7"N 99°05'20.4"E | 2 |
| M024 | Tham Malai, Phattalung | *S. dehaani* | 00264 | 7°38'58.3"N 100°06'23.7"E | 1 |
| M025 | Kao Sok Resort, Kaosok, Suratthani | *S. dehaani* | 00265 | 8°54'18.9"N 98°31'21.1"E | 1 |
| M026 | Kham Hom Waterfall, Sakon Nakhorn | *S. dehaani* | 00266 | 17°07'19.9"N 104°01'07.4"E | 1 |
| M027 | Wat Huai To, Mueang, Krabi | *S. dehaani* | 00267 | 8°13'37.6"N 98°53'01.7"E | 1 |
| M028 | Wat Namtok, Saiyok, Kanchanaburi | *S. dehaani* | 00268 | 14°13'47.5"N 99°03'59.8"E | 1 |
| M029 | Samet Island, Ban Phe, Rayong | *S. dehaani* | 00269 | 12°34'04.3"N 101°27'23.3"E | 3 |
| M030 | Wat Tham Lijia, Sangkhaburi, Kanchanaburi | *S. dehaani* | 00270 | 15°04'12.6"N 98°33'59.0"E | 1 |
| M031 | Wat Tham Phupha, Thakantho, Kalasin | *S. dehaani* | 00271 | 16°48'05.7"N 103°12'37.5"E | 1 |
| M032 | Saphan Hin Waterfall, Khlong Yai, Trad | *S. dawydoffi* | 00272 | 12°06'06.0"N 102°42'39.2"E | 2 |
| M033 | Khao Phlai Dam, Sichon, Nakhon Sri Thammarat | *S. dehaani* | 00273 | 9°05'33.4"N 99°54'25.0"E | 2 |
| M034 | Tham Wang Thong, Khuan Kanun, Phattalung | *S. dehaani* | 00274 | 7°40'55.1"N 100°00'56.8"E | 5 |
| M035 | Ban Thatoom, Mahasarakam | *S. dehaani* | 00275 | 16°10'32.2"N 103°26'59.6"E | 3 |
| M036 | Wat Tham Erawan, Ban Rai, Uthaithani | *S. dehaani* | 00276 | 15°02'01.5"N 99°27'16.6"E | 1 |
| M037 | Tham Pha Pu, Loei | *S. dehaani* | 00277 | 17°34'41.5"N 101°42'39.1"E | 2 |
| M038 | Bok Krai Waterfall, Kraburi, Ranong | *S. dehaani* | 00278 | 10°22'34.6"N 98°51'18.3"E | 1 |
| M039 | Nang Rong Waterfall, Nakhon Nayok | *S. dehaani* | 00279 | 14°19'52.5"N 101°19'09.1"E | 1 |
| M040 | Tham Su Mano, Srinakarin, Phattalung | *S. dehaani* | 00280 | 7°35'12.3"N 99°52'04.3"E | 1 |
| M041 | Klong Phot Waterfall, Nop Phitam, Nakhon Sri Thammarat | *S. dehaani* | 00281 | 7°48'37.8"N 99°12'20.0"E | 2 |
| M042 | Wang Kanlueang Waterfall, Chai Badan, Lopburi | *S. dehaani* | 00282 | 15°06'49.4"N 101°06'38.8"E | 1 |
| M043 | Rog Nai Island, Koh Lanta, Krabi | *S. dehaani* | 00283 | 7°13'12.7"N 99°04'12.7"E | 10 |
| M044 | Pa Son Nongkhu, Sangkhla, Surin | *S. dehaani* | 00284 | 14°40'55.7"N 103°45'51.9"E | 1 |
| M045 | Juang Island, Sattahip, Chonburi | *S. dehaani* | 00285 | 12°31'46.4"N 100°57'18.4"E | 1 |
| M046 | Chaloemphrakiat, Saraburi,Thailand | *S. dehaani* | 00286 | 14°40'11.9"N 100°53'09.4"E | 1 |
| M047 | Ban Phon Thong, Kaset Wisai, Roi-Et | *S. dehaani* | 00287 | 15°39'59.6"N 103°33'10.9"E | 1 |
| M048 | Phusang Waterfall, Phusang, Phayao | *S. dehaani* | 00288 | 19°40'05.0"N 100°23'25.1"E | 1 |
| M049 | Wat Khao Somphot, Chai Badan, Lopburi | *S. dehaani* | 00289 | 15°09'42.2"N 101°16'49.5"E | 1 |
| M050 | Sakaerat, Wang Namkhiao, Nakhon Ratchasima | *S. dawydoffi* | 00290 | 14°30'36.5"N 101°55'51.5"E | 1 |
| M051 | Wat Khao Sarp, Rayong | *S. dehaani* | 00291 | 12°36'46.8"N 101°23'18.8"E | 1 |
| M052 | Wat Tham Pak Khaew Chiang Khan, Loei | *S. dehaani* | 00292 | 17°52'34.0"N 101°40'20.8"E | 1 |
| M053 | Tham Khao Kriab, Pathio, Chumporn | *S. dehaani* | 00293 | 9°49'01.3"N 99°02'17.9"E | 1 |
| M054 | Wat Thang Biang, Pak Chong, Nakhon Ratchasima | *S. dawydoffi* | 00294 | 14°32'22.0"N 101°21'54.6"E | 2 |
| M055 | Tat E-tu, Paksong, Champasak, Laos | *Scolopendra* sp. | 00295 | 15°13'10.6"N 105°55'31.3"E | 1 |
| M056 | Tat Pha Yueang, Mueang Sing, Luang Namtha, Laos | *Scolopendra* sp. | 00296 | 15°09'55.1"N 106°06'10.6"E | 1 |
| M057 | Phu Fah Mountain, Phongsali, Laos | *S. japonica* | 00297.1-3 | 21°41'19.6"N 102°06'30.4"E | 3 |
| M058 | Plain of Jar, Xiang Khouang, Laos | *S. japonica* | 00298 | 19°25'51.5"N 103°09'10.4"E | 5 |
| M059 | Wang Bua, Kabin Buri, Prachinburi | *S. morsitans* | 00299 | 13°57'16.3"N 101°36'37.3"E | 1 |
| M060 | Mueang, Sakaeo | *S. morsitans* | 00300 | 13°49'07.9"N 102°03'10.5"E | 4 |
| M061 | Kuiburi, Prachuab Khirikhan | *S. morsitans* | 00301 | 12°06'32.0"N 99°45'53.0"E | 1 |
| M062 | Wat Mahavanh, Buriram | *S. morsitans* | 00302 | 14°41'09.8"N 102°52'33.8"E | 4 |
| M063 | Wat Tham Lijia, Sangkhlaburi, Kanchanaburi | *S. pinguis* | 00303 | 15°04'12.8"N 98°33'56.4"E | 1 |
| M064 | Wiang Thong Hot spring, Mueang Iam, Huaphan, Laos | *S. pinguis* | 00304 | 20°04'45.2"N 103°44'33.3"E | 1 |
| M065 | Phusang Waterfall, Phusang Phayao | *S. pinguis* | 00305 | 19°37'10.2"N 100°21'54.7"E | 1 |
| M066 | Ban Na-Ton, Laos | *S. pinguis* | 00306 | 17°52'31.4"N 104°51'44.7"E | 2 |
| M067 | Huai Nam Un, Nan | *S. pinguis* | 00307 | 18°30'22.8"N 100°31'49.1"E | 1 |
| M068 | Ban Krung Klai, Laos | *S. pinguis* | CUMZ 00308 | 20°24'29.7"N 100°51'45.8"E | 1 |
| M069 | Kao Rao Cave, Bokaeow, Laos | *S. pinguis* | CUMZ 00309 | 20°41'56.6"N 101°05'46.8"E | 1 |
| M070 | Kra Cham Waterfall, Luang Prabang, Laos | *S. pinguis* | CUMZ 00310 | 19°32'27.3"N 101°59'02.3"E | 2 |
| M071 | Tat Ton Waterfall, Chaiyaphum | *S. pinguis* | CUMZ 00311 | 16°01'05.2"N 102°01'24.4"E | 1 |
| M072 | Chong Khao Khad, Saiyok, Kanchanaburi | *S. pinguis* | CUMZ 00312 | 14°22'47.6"N 98°55'47.7"E | 1 |
