# Supplementary material for: The Centipede Genus Scolopendra in Mainland Southeast Asia: Molecular Phylogenetics, Geometric Morphometrics and External Morphology as Tools for Species Delimitation
Source: PLoS One. 2015 Aug 13;10(8):e0135355. doi: 10.1371/journal.pone.0135355 (PMC4536039; doi:10.1371/journal.pone.0135355)
Supplement: S2 Table — (DOCX) [file pone.0135355.s003.docx]

**S2 Table**

| **Character** | **Centroid size** | | | **Shape** | | |
| --- | --- | --- | --- | --- | --- | --- |
|  | df | f | *P*-value | df | f | *P*-value |
| Cephalic plate | 5 | 19.52 | <0.001 | 90 | 2.96 | <0.001 |
| Coxosternite | 5 | 16.35 | <0.001 | 110 | 2.28 | <0.001 |
| Tergite 21 | 5 | 14.89 | <0.001 | 70 | 34.11 | <0.001 |

df-degrees of freedom, *F*- *F* statistic value and *p*- *p* statistical significance value (p-values below 0.0001 suggest no significant error from landmark acquisition in each analysis)
