# Supplementary material for: The Centipede Genus Scolopendra in Mainland Southeast Asia: Molecular Phylogenetics, Geometric Morphometrics and External Morphology as Tools for Species Delimitation
Source: PLoS One. 2015 Aug 13;10(8):e0135355. doi: 10.1371/journal.pone.0135355 (PMC4536039; doi:10.1371/journal.pone.0135355)
Supplement: S3 Table — (DOCX) [file pone.0135355.s004.docx]

**S3 Table**

| **CV** | **Cephalic plate** | | **Coxosternite** | | **Tergite 21** | |
| --- | --- | --- | --- | --- | --- | --- |
|  | **Eigenvalues** | **% variance** | **Eigenvalues** | **% variance** | **Eigenvalues** | **% variance** |
|  |  |  |  |  |  |  |
| 1 | 1.5234 | 46.468 | 4.0351 | 54.298 | 4.5012 | 64.358 |
| 2 | 0.8612 | 26.269 | 2.2834 | 30.728 | 1.8013 | 25.756 |
| 3 | 0.5106 | 15.575 | 0.591 | 7.995 | 0.3887 | 5.558 |
| 4 | 0.2489 | 7.594 | 0.3278 | 4.412 | 0.1889 | 2.701 |
| 5 | 0.1341 | 4.093 | 0.1907 | 2.567 | 0.1137 | 1.627 |

The first column represents CV 1 to CV 5 axes while each row indicates the eigenvalue and percentage of variance in each examined feature.
