# Supplementary material for: The Centipede Genus Scolopendra in Mainland Southeast Asia: Molecular Phylogenetics, Geometric Morphometrics and External Morphology as Tools for Species Delimitation
Source: PLoS One. 2015 Aug 13;10(8):e0135355. doi: 10.1371/journal.pone.0135355 (PMC4536039; doi:10.1371/journal.pone.0135355)
Supplement: S4 Table — (DOCX) [file pone.0135355.s005.docx]

**S4 Table**

|  | **Taxon** | **Mahalanobis distances** | | | | | |
| --- | --- | --- | --- | --- | --- | --- | --- |
|  |  | *S. dawydoffi* | *S. dehaani* | *S. japonica* | *S. morsitans* | *S. pinguis* | *Scolopendra* sp. |
| **Procrustes distance** | *S. dawydoffi* |  | 2.374 (0.0406) | 4.4967 (0.0003) | 3.8486 (0.0014) | 2.8143 (0.3411) | 4.7970 (0.0208) |
|  | *S. dehaani* | 0.0435 (0.0936) |  | 4.5332  (<0.0001) | 3.3998 (<0.0001) | 2.2191 (<0.0001) | 5.3307 (0.0002) |
|  | *S. japonica* | 0.054 (0.0198) | 0.0456 (0.0186) |  | 4.5167 (<0.0001) | 3.8673 (0.0002) | 5.5612 (0.0068) |
|  | *S. morsitans* | 0.064 (0.0021) | 0.0354 (0.0488) | 0.0498 (0.0073) |  | 4.0926 (<0.0001) | 5.7660 (0.0019) |
|  | *S. pinguis* | 0.0243 (0.6426) | 0.0418 (0.0120) | 0.0500 (0.0071) | 0.0656 (0.0006) |  | 5.8302 (0.0139) |
|  | *Scolopendra* sp. | 0.0288 (0.7002) | 0.0429 (0.3707) | 0.0465 (0.4971) | 0.0601 (0.0874) | 0.0321 (0.8267) |  |

*p*- statistic values (in parentheses) below 0.0001 indicate significant distinctness of two classifiers.
