# Supplementary material for: The Centipede Genus Scolopendra in Mainland Southeast Asia: Molecular Phylogenetics, Geometric Morphometrics and External Morphology as Tools for Species Delimitation
Source: PLoS One. 2015 Aug 13;10(8):e0135355. doi: 10.1371/journal.pone.0135355 (PMC4536039; doi:10.1371/journal.pone.0135355)
Supplement: S5 Table — (DOCX) [file pone.0135355.s006.docx]

**S5 Table**

|  | **Taxon** | **Mahalanobis distances** | | | | | |
| --- | --- | --- | --- | --- | --- | --- | --- |
|  |  | *S. dawydoffi* | *S. dehaani* | *S. japonica* | *S. morsitans* | *S. pinguis* | *Scolopendra* sp. |
| **Procrustes distance** | *S. dawydoffi* |  | 3.9370 (<0.0001) | 4.2097 (0.0004) | 4.4170 (0.0004) | 6.3117 (0.0001) | 6.6842 (0.0401) |
|  | *S. dehaani* | 0.0527 (0.0225) |  | 3.9956  (<0.0001) | 6.4878 (<0.0001) | 5.1480 (<0.0001) | 4.8350 (0.0005) |
|  | *S. japonica* | 0.0452 (0.0529) | 0.0350 (0.0648) |  | 4.9057 (<0.0001) | 5.1750 (<0.0001) | 6.4816 (0.0024) |
|  | *S. morsitans* | 0.0506 (0.0132) | 0.0695 (<0.0001) | 0.0553 (0.0002) |  | 6.9341 (0.0079) | 9.4402 (<0.0001) |
|  | *S. pinguis* | 0.0655 (0.0434) | 0.0743 (<0.0001) | 0.0651 (0.0073) | 0.1067 (<0.0001) |  | 7.7283 (0.0083) |
|  | *Scolopendra* sp. | 0.0796 (0.0317) | 0.0492 (0.2810) | 0.0662 (0.1007) | 0.0927 (0.0114) | 0.0759 (0.1312) |  |

*p*- statistic values (in parentheses) below 0.0001 indicate significant distinctness of two classifiers.
