# Supplementary material for: The Centipede Genus Scolopendra in Mainland Southeast Asia: Molecular Phylogenetics, Geometric Morphometrics and External Morphology as Tools for Species Delimitation
Source: PLoS One. 2015 Aug 13;10(8):e0135355. doi: 10.1371/journal.pone.0135355 (PMC4536039; doi:10.1371/journal.pone.0135355)
Supplement: S6 Table — (DOCX) [file pone.0135355.s007.docx]

**S6 Table**

|  | **Taxon** | **Mahalanobis distances** | | | | | |
| --- | --- | --- | --- | --- | --- | --- | --- |
|  |  | *S. dawydoffi* | *S. dehaani* | *S. japonica* | *S. morsitans* | *S. pinguis* | *Scolopendra* sp. |
| **Procrustes distance** | *S. dawydoffi* |  | 4.9933 (<0.0001) | 3.1822 (0.0094) | 3.0515 (0.0396) | 5.3469 (<0.0001) | 8.5261 (0.0477) |
|  | *S. dehaani* | 0.1519 (<0.0001) |  | 4.2159 (<0.0001) | 6.3377 (<0.0001) | 4.4531 (<0.0001) | 4.4531 (0.0013) |
|  | *S. japonica* | 0.0556 (0.0300) | 0.1065 (<0.0001) |  | 3.5160 (0.0002) | 5.1449 (<0.0001) | 7.0169 (0.0105) |
|  | *S. morsitans* | 0.0319 (0.1989) | 0.1776 (<0.0001) | 0.0760 (0.0008) |  | 6.9018 (<0.0001) | 9.3133 (0.0061) |
|  | *S. pinguis* | 0.0552 (0.0355) | 0.01336 (<0.0001) | 0.0704 (0.0003) | 0.0788 (<0.0001) |  | 7.2634 (0.0097) |
|  | *Scolopendra* sp. | 0.2394 (0.0206) | 0.0928 (0.0120) | 0.1899 (0.0218) | 0.2631 (0.0034) | 0.2242 (0.0039) |  |

*p*- statistic values (in parentheses) below 0.0001 indicate significant distinctness of two classifiers
